# Women's Access and Provider Practices for the Case Management of Malaria during Pregnancy: A Systematic Review and Meta-Analysis

**DOI:** 10.1371/journal.pmed.1001688

**Published:** 2014-08-05

**Authors:** Jenny Hill, Lauren D'Mello-Guyett, Jenna Hoyt, Anna M. van Eijk, Feiko O. ter Kuile, Jayne Webster

**Affiliations:** 1 Department of Clinical Sciences, Liverpool School of Tropical Medicine, Liverpool, United Kingdom; 2 Disease Control Department, London School of Hygiene & Tropical Medicine, London, United Kingdom; Hospital Clinic Barcelona, Spain

## Abstract

**Background:**

WHO recommends prompt diagnosis and quinine plus clindamycin for treatment of uncomplicated malaria in the first trimester and artemisinin-based combination therapies in subsequent trimesters. We undertook a systematic review of women's access to and healthcare provider adherence to WHO case management policy for malaria in pregnant women.

**Methods and Findings:**

We searched the Malaria in Pregnancy Library, the Global Health Database, and the International Network for the Rational Use of Drugs Bibliography from 1 January 2006 to 3 April 2014, without language restriction. Data were appraised for quality and content. Frequencies of women's and healthcare providers' practices were explored using narrative synthesis and random effect meta-analysis. Barriers to women's access and providers' adherence to policy were explored by content analysis using NVivo. Determinants of women's access and providers' case management practices were extracted and compared across studies. We did not perform a meta-ethnography. Thirty-seven studies were included, conducted in Africa (30), Asia (4), Yemen (1), and Brazil (2). One- to three-quarters of women reported malaria episodes during pregnancy, of whom treatment was sought by >85%. Barriers to access among women included poor knowledge of drug safety, prohibitive costs, and self-treatment practices, used by 5%–40% of women. Determinants of women's treatment-seeking behaviour were education and previous experience of miscarriage and antenatal care. Healthcare provider reliance on clinical diagnosis and poor adherence to treatment policy, especially in first versus other trimesters (28%, 95% CI 14%–47%, versus 72%, 95% CI 39%–91%, *p* = 0.02), was consistently reported. Prescribing practices were driven by concerns over side effects and drug safety, patient preference, drug availability, and cost. Determinants of provider practices were access to training and facility type (public versus private). Findings were limited by the availability, quality, scope, and methodological inconsistencies of the included studies.

**Conclusions:**

A systematic assessment of the extent of substandard case management practices of malaria in pregnancy is required, as well as quality improvement interventions that reach all providers administering antimalarial drugs in the community. Pregnant women need access to information on which anti-malarial drugs are safe to use at different stages of pregnancy.

*Please see later in the article for the Editors' Summary*

## Abstract 2

Jenny Hill and colleagues conduct a systematic review and meta-analysis of womenâ€™s access and healthcare provider adherence to WHO case-management policy of malaria during pregnancy.

*Please see later in the article for the Editors' Summary*

## Editors' Summary

**Background:**

Malaria, a mosquito-borne parasite, kills about 600,000 people every year. Most of these deaths occur among young children in sub-Saharan Africa, but pregnant women and their unborn babies are also vulnerable to malaria. Infection with malaria during pregnancy can cause severe maternal anemia, miscarriages, and preterm births, and kills about 10,000 women and 100,000 children each year. Since 2006, the World Health Organization (WHO) has recommended that uncomplicated malaria (an infection that causes a fever but does not involve organ damage or severe anemia) should be treated with quinine and clindamycin if it occurs during the first trimester (first three months) of pregnancy and with an artemisinin-based combination therapy (ACT) if it occurs during the second or third trimester; ACTs should be used during the first trimester only if no other treatment is immediately available because their safety during early pregnancy has not been established. Since 2010, WHO has also recommended that clinical diagnosis of malaria should be confirmed before treatment by looking for parasites in patients' blood (parasitology).

**Why Was This Study Done?:**

Prompt diagnosis and treatment of malaria in pregnancy in regions where malaria is always present (endemic regions) is extremely important, yet little is known about women's access to the recommended interventions for malaria in pregnancy or about healthcare providers' adherence to the WHO case management guidelines. In this systematic review and meta-analysis of qualitative, quantitative, and mixed methods studies, the researchers explore the factors that affect women's access to treatment and healthcare provider practices for case management of malaria during pregnancy. A systematic review uses predefined criteria to identify all the research on a given topic. Meta-analysis is a statistical method for combining the results of several studies. A qualitative study collects non-quantitative data such as reasons for refusing an intervention, whereas a qualitative study collects numerical data such as the proportion of a population receiving an intervention.

**What Did the Researchers Do and Find?:**

The researchers identified 37 studies (mostly conducted in Africa) that provided data on the range of healthcare providers visited, antimalarials used, and the factors influencing the choice of healthcare provider and medicines among pregnant women seeking treatment for malaria and/or the type and quality of diagnostic and case management services offered to them by healthcare providers. The researchers explored the data in these studies using narrative synthesis (which summarizes the results from several qualitative studies) and content analysis (which identifies key themes within texts). Among the studies that provided relevant data, one-quarter to three-quarters of women reported malaria episodes during pregnancy. More than 85% of the women who reported a malaria episode during pregnancy sought some form of treatment. Barriers to access to WHO-recommended treatment among women included poor knowledge about drug safety, and the use of self-treatment practices such as taking herbal remedies. Factors that affected the treatment-seeking behavior of pregnant women (“determinants”) included prior use of antenatal care, education, and previous experience of a miscarriage. Among healthcare providers, reliance on clinical diagnosis of malaria was consistently reported, as was poor adherence to the treatment policy. Specifically, 28% and 72% of healthcare providers followed the treatment guidelines for malaria during the first and second/third trimesters of pregnancy, respectively. Finally, the researchers report that concerns over side effects and drug safety, patient preference, drug availability, and cost drove the prescribing practices of the healthcare providers, and that the determinants of provider practices included the type (cadre) of heathcare worker, access to training, and whether they were based in a public or private facility.

**What Do These Findings Mean?:**

These findings reveal important limitations in the implementation of the WHO policy on the treatment of malaria in pregnancy across many parts of Africa and in several other malaria endemic regions. Notably, they show that women do not uniformly seek care within the formal healthcare system and suggest that, when they do seek care, they may not be given the appropriate treatment because healthcare providers frequently fail to adhere to the WHO diagnostic and treatment guidelines. Although limited by the sparseness of data and by inconsistencies in study methodologies, these findings nevertheless highlight the need for further systematic assessments of the extent of substandard case management of malaria in pregnancy in malaria endemic countries, and the need to develop interventions to improve access to and delivery of quality case management of malaria among pregnant women.

**Additional Information:**

Please access these websites via the online version of this summary at http://dx.doi.org/10.1371/journal.pmed.1001688.

- Information is available from the World Health Organization on malaria (in several languages) and on malaria in pregnancy; the 2010 Guidelines for the Treatment of Malaria are available; the World Malaria Report 2013 provides details of the current global malaria situation
- The US Centers for Disease Control and Prevention also provides information on malaria; a personal story about malaria in pregnancy is available
- Information is available from the Roll Back Malaria Partnership on all aspects of global malaria control, including information on malaria in pregnancy
- The Malaria in Pregnancy Consortium is undertaking research into the prevention and treatment of malaria in pregnancy and provides links to the consortium's publications and an online library on malaria in pregnancy
- MedlinePlus provides links to additional information on malaria (in English and Spanish)

## Introduction

Malaria in pregnancy is an important public health problem for both maternal and neonatal health programmes. The manifestation of maternal infection with malaria depends on transmission intensity, and prompt diagnosis and treatment of malaria illness in pregnancy is important in all malaria endemic regions. Since 2006, WHO recommends quinine plus clindamycin for the treatment of uncomplicated malaria in the first trimester, and artesunate (AS) plus clindamycin for treatment failures. Artemisinin-based combination therapies (ACTs) known to be effective in the country/region, or AS plus clindamycin, are the recommended combinations for case management of uncomplicated malaria in the second and third trimesters [1],[2]. Use of the artemisinin class of compounds, alone or in combination therapies, is not recommended in the first trimester of pregnancy because of insufficient safety data in early pregnancy in humans [3], unless this is the only treatment immediately available [1].

Many countries in high transmission settings have made ACTs available free of charge to pregnant women in efforts to achieve universal coverage [4]. Despite increasing availability of ACTs and new diagnostic tools, such as rapid diagnostic tests (RDTs), very little is known about women's access to these interventions and about the diagnosis and treatment practices of healthcare providers. National malaria indicator surveys focus on access to case management among children, the other important risk group for malaria. Similarly, research on uptake of new diagnostics and ACTs has to date focussed on children and non-pregnant adult populations, whereas research on uptake of interventions in pregnancy has predominantly focussed on progress and challenges to the delivery and uptake of preventive interventions, namely, intermittent preventive treatment in pregnancy (IPTp) and insecticide-treated nets [5]. Information on access to and delivery of effective case management of malaria in pregnancy has not yet received the attention it deserves.

We undertook a systematic review of the factors affecting pregnant women's access to and health provider adherence to the 2006 WHO policy [2] on the treatment of malaria in pregnancy globally. Among pregnant women we reviewed treatment-seeking practices for malaria illness—the range of providers visited, the antimalarials used, and the factors affecting their choice of healthcare provider and medicines. We explored adherence to policy among the range of healthcare providers administering antimalarials to pregnant women, the type and quality of diagnostic and case management services offered at the point of care (including consideration of gestational age), and the health system or other factors that affect quality of care.

## Methods

### Search Strategy

Studies investigating treatment-seeking practices for malaria among pregnant women and healthcare provider case management practices for malaria in pregnancy were identified by searching the Malaria in Pregnancy Library [6], the Global Health Database [7], and the International Network for the Rational Use of Drugs (INRUD) Bibliography [8] from 1 January 2006 to 3 April 2014. The Malaria in Pregnancy Library (http://library.mip-consortium.org) is a comprehensive bibliographic database created by the Malaria in Pregnancy Consortium that is updated every 4 mo using a standardised protocol to search over 40 sources, including PubMed, Web of Knowledge, and Google Scholar. Searches were run separately for “pregnant women” and “health providers” (see Table S1 for search terms), without language restrictions, and both peer-reviewed and grey literature were retrieved.

### Study Selection

Titles and abstracts were reviewed independently for inclusion by two reviewers (J. Hill and L. D′M-G/J. Hoyt). Studies were included if they met the following criteria: (1) study contained data on treatment seeking among women and/or case management practices for malaria in pregnancy, (2) study population included pregnant women and/or healthcare providers, (3) study reported original research data, and (4) study was conducted following the introduction of ACTs for the treatment of uncomplicated malaria in pregnancy in the study country. No restrictions were placed on study design (i.e., quantitative, qualitative, and mixed methods studies), or quality of reporting. Studies limited to knowledge of malaria in pregnancy amongst pregnant women, i.e., without information on practices, were excluded. The Kappa (K) statistic was used as a measure of the inter-rater agreement on study eligibility between reviewers. Discrepancies between reviewers were resolved through discussions with a third reviewer (J. W.) until consensus was reached.

Studies meeting the inclusion criteria were assessed and grouped according to content. Among pregnant women primary outcomes included (1) treatment-seeking practices for malaria, (2) barriers to accessing malaria treatment, and (3) determinants of treatment seeking for malaria. Among healthcare providers primary outcomes were (1) case management practices for malaria in pregnancy, (2) factors affecting malaria case management practices, and (3) determinants of knowledge, diagnosis, and treatment of malaria.

### Data Extraction

**Figure 1:**
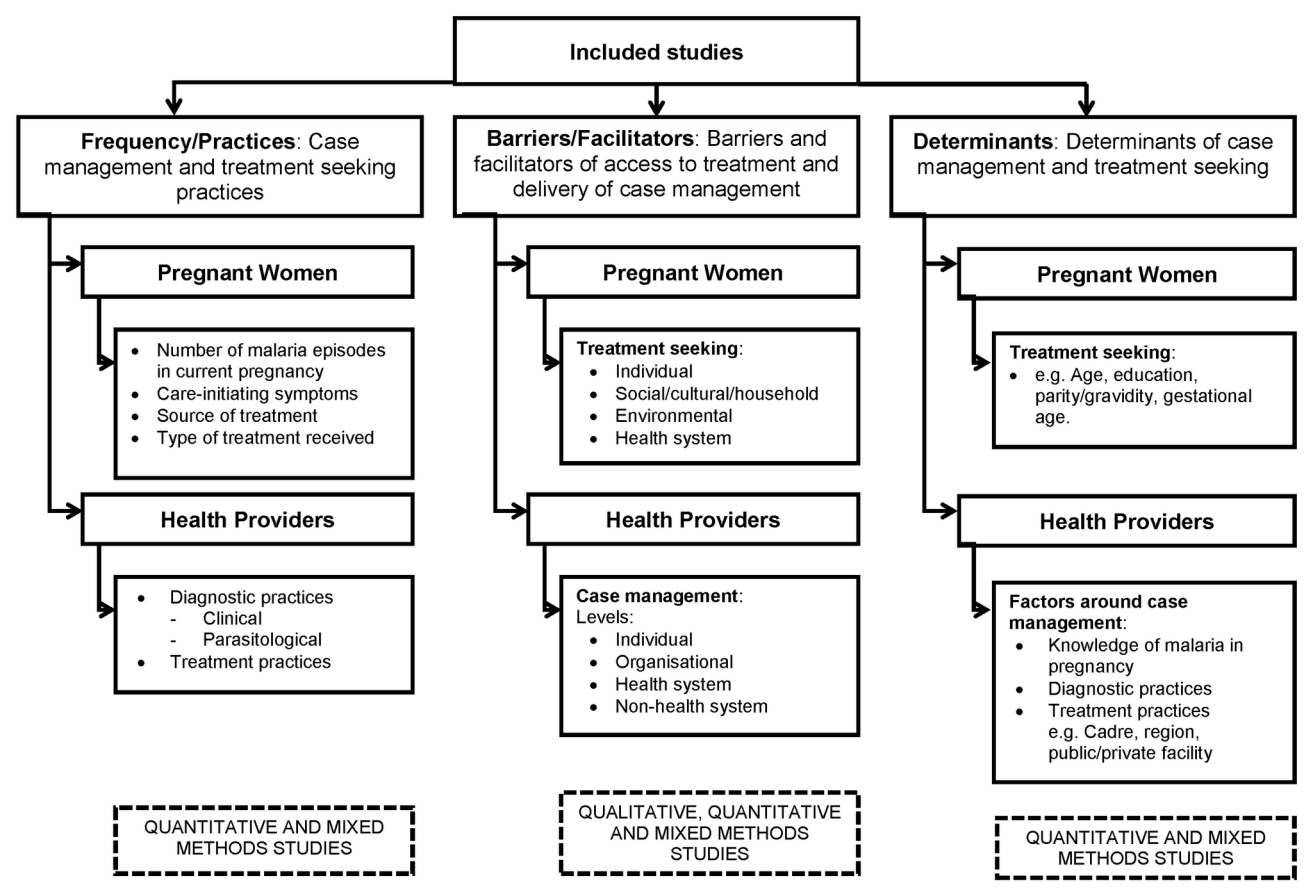
Analysis strategy.

Two authors extracted data and appraised the quality and content of included studies. Data for pregnant women or healthcare providers were extracted and analysed separately for description and frequency of practices, barriers/facilitators, and determinants (Figure 1). Two authors (J. Hill and L. D′M-G/A. M. v. E/J. Hoyt) extracted quantitative data on the type and frequency of practices from quantitative and mixed methods studies. For pregnant women these quantitative data included the frequency of malaria episodes, sources of treatment, and the resultant treatment achieved, and for healthcare providers the quantitative data included the type and frequency of diagnostic and treatment practices in relation to national drug policy at the time of publication. J. Hill and L. D′M-G/J. Hoyt extracted qualitative and quantitative data on the barriers and facilitators to treatment seeking among pregnant women and case management practices among healthcare providers from qualitative and mixed methods studies. J. Hill and L. D′M-G/J. Hoyt extracted quantitative data on the determinants of treatment seeking and case management practices among pregnant women and healthcare providers, respectively, from quantitative and mixed methods studies. For healthcare providers, determinants of knowledge and practice, and of diagnosis and treatment, were extracted separately. Two authors (J. Hill and L. D′M-G/J. Hoyt) assessed the quality of reporting of individual studies using a checklist of criteria developed a priori based on criteria and methods described in the literature, described previously [5].

### Data Synthesis and Analysis

Narrative synthesis was used to summarise, compare, and contrast the type, range, and frequency of practices from each study evaluating treatment seeking among pregnant women and case management practices among healthcare providers. To make a comparison between national policy and healthcare provider practices by country and region, we used the national or global malaria policy cited in the included studies.

Barriers and facilitators were explored using content analysis with a previously defined thematic framework for pregnant women and healthcare providers [5]. NVivo version 9.2 (QSR International) was used to generate an index of codes, which identified each of the recurring barriers amongst pregnant women and healthcare providers. The themes emerged as all the data were analysed, working cyclically through the studies. Data from the women's perspective were categorised into individual, social/cultural/household, environmental, and health system levels. Data from providers were synthesised into a matrix that combined operational levels of individual, organisational, health system, and non-health system levels, together with the six health systems levels of the WHO Health Systems Framework, which include governance/leadership, service delivery, health workforce/human resources, health information systems, finance, and medical products/technologies [9]–[11].

We appraised the quality of reporting of each study using a checklist of criteria based on methods described in a previous review [5], as described and reported in Tables S2–S4.

### Statistical Analysis

We pooled the frequency data for source of treatment among pregnant women and adherence to treatment policy among healthcare providers across different types of providers using random effect meta-analysis in Stata version 12 (StataCorp) and Comprehensive Meta-Analysis (Biostat; http://www.meta-analysis.com/), which was also used for sub-group analysis. We used forest plots to visualise the extent of heterogeneity between studies. For studies that reported source of treatment for more than one episode of fever, we included the response to the first episode [12]. For source of treatment among pregnant women, we conducted sub-group analysis within each category for the following: whether the question involved practice (i.e., women with fever) or attitude (i.e., a hypothetical question, “if they had fever…”); health facility– or population-based enrolment; urban or rural populations; and country of study (Nigeria, the country contributing the majority of studies, versus other countries). For adherence to treatment policy, we conducted sub-group analysis for the following: trimester treated, the effect of staff cadre (medical doctor versus others), and method of data collection (self-administered questionnaire, interview, or record review). *I* ^2^ was used to quantify heterogeneity [13].

## Results

**Figure 2:**
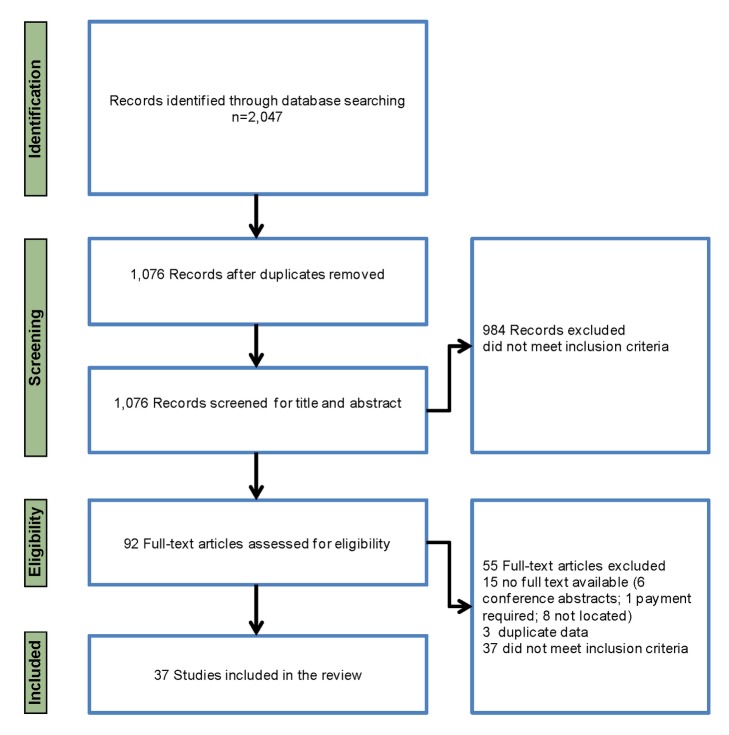
PRISMA chart of studies included in the review.

Of 2,047 records retrieved from the database searches, 37 studies met the inclusion criteria (Figure 2)—13 studies in pregnant women, 18 studies in healthcare providers, and six studies in both pregnant women and healthcare providers; only one study evaluated interventions. There was close agreement between the reviewers on the review of full text articles (K = 0.84). The majority of studies were conducted in Africa (30), with only four studies conducted in Asia (two in India [14],[15] and two in Cambodia [16],[17]), one in Yemen [18], and two in Brazil [19],[20]. Of the studies conducted in Africa, 17 were in west and central Africa and 12 in east and southern Africa, and one study had sites in east, west and southern Africa [21]. All but three studies were cross-sectional surveys at the population or facility level. The remaining studies included two longitudinal qualitative studies [21],[22] and a randomised controlled trial [23]. The study characteristics are provided in Tables 1–3.

**Table 1:** Characteristics of studies reporting outcomes, barriers, and determinants for treatment-seeking practices among pregnant women (13 studies).

Study Description											Primary Outcomes		
Study	Region	Country	Scale	Urban/Rural	Study Year	Target Population	Study Design	Data Type	Sample	*N*	Treatment-Seeking Practices	Barriers to Treatment Seeking	Determinants of Treatment Seeking
Adam 2008 [31]	East Africa	Sudan	1 district	Rural	2006	Population	Cross-sectional	Quantitative	PW	168	√	√	
Enato 2009 [27]	West Africa	Nigeria	1 state	Urban	2005	Facility	Cross-sectional	Quantitative	PW	630	√	√	
Henry 2012 [26]	East Africa	Uganda	1 district	IDP camps	2007–2008	Population	Cross-sectional	Quantitative	PW	769	√		√
Karunamoorthi 2010 [32]	East Africa	Ethiopia	<1 district	Urban	2008	Facility	Cross-sectional	Quantitative	PW	225	√	√	
Launiala 2010 [22]	Southern Africa	Malawi	<1 district	Rural	2002 + 2006	Facility	Longitudinal	Qualitative	Women/PW	34/8	√	√	
Maiga 2010 [12]	West Africa	Mali	<1 district	Rural	—	Facility	Cross-sectional	Quantitative	PW	210	√	√	
Mbachu 2012 [24]	West Africa	Nigeria	1 district	Rural	2011	Population	Cross-sectional	Quantitative	PW	898	√	√	√
Mbonye 2013 [34]	East Africa	Uganda	1 district	Rural/urban	2011	Facility	Cross-sectional	Quantitative	PW	998	√		
Onwujekwe 2013 [29]	West Africa	Nigeria	<1 district	Urban	—	Facility	Cross-sectional	Quantitative	PW	647	√		
Sabin 2010 [14]	Asia	India	1 state	Rural/urban	2007	Facility	Cross-sectional	Mixed	PW/RD 12	73	√	√	
Sam-Wobo 2008 [33]	West Africa	Nigeria	1 district	Rural/urban	2006	Population	Cross-sectional	Quantitative	PW	1,400	√	√	
Sangaré 2011 [25]	East Africa	Uganda	<1 district	Rural/urban	2008–2009	Population	Cross-sectional	Quantitative	PW	500	√	√	√
Smith Paintain 2010 [23]	West Africa	Ghana	2 districts	Rural	2009	Facility	RCT	Qualitative	PW	1,486		√	

PW, pregnant women; RCT, randomised controlled trial; RD 12, recently delivered women (last 12 mo).

**Table 2:** Characteristics of studies reporting outcomes, barriers, and determinants for case management practices among healthcare providers (18 studies).

Study Description											Primary Outcomes			
Study	Region	Country	Scale	Urban/Rural	Study Year	Target Population	Study Design	Data Type	Sample	*N*	Knowledge and Practices		Barriers to Case Management	Determinants of Case Management
											Diagnostics	Treatment		
Enato 2012 [44]	West Africa	Nigeria	<1 district	Urban	—	Population	Cross-sectional	Qualitative	TBA	8	√	√	√	
Bin Ghouth 2013 [18]	Middle East	Yemen	11 districts	Urban	2010–2011	Facility	Before-after	Quantitative	Pharm/HP	86		√	√	√
Harrison 2012 [43]	West Africa	Nigeria	1 district	Urban	2009	Facility	Cross-sectional	Quantitative	MD	123	√	√	√	√
Kalilani-Phiri 2011 [42]	Southern Africa	Malawi	National	Rural/urban	2010	Population	Cross-sectional	Quantitative	MD/pharm	92	√		√	
Kiningu 2013 [41]	East Africa	Kenya	<1 district	Urban	2012	Facility	Cross-sectional	Mixed	MD/nurse/pharm	36	√	√	√	√
Luz 2013 [20]	South America	Brazil	1 district	Urban	2007–2008	Facility	Cross-sectional	Mixed	MD/nurse/pharm	51		√	√	
Luz 2013 [19]	South America	Brazil	>1 district	Urban	2007–2008	Facility	Cross-sectional	Quantitative	PW+HP§	262		√		√
Minyaliwa 2012 [51]	Southern Africa	Malawi	1 district	Urban	—	Facility	Cross-sectional	Quantitative	Pharma/nurse/pharma technician	22		√	√	
Okonta 2011 [46]	West Africa	Nigeria	National	Rural/urban	2008	Population	Cross-sectional	Quantitative	MD	102		√	√	
Okoro 2012 [40]	West Africa	Nigeria	<1 district	Urban	2009	Facility	Cross-sectional	Quantitative	MD	311	√	√	√	
Omo-Aghoja 2008 [37]	West Africa	Nigeria	National	Rural/urban	2006	Facility	Cross-sectional	Quantitative	MD	84	√	√	√	√
Onwujekwe 2012 [39]	West Africa	Nigeria	<1 district	Urban	2010	Facility	Cross-sectional	Quantitative	MD/nurse/pharm	52	√	√	√	√
PSI 2007 [16]	Asia	Cambodia	National	Rural/urban	2007	Facility	Cross-sectional	Mixed	MD/MA/pharm/nurse/midwife/DV	750	√		√	
Smith Paintain 2011 [47]	West Africa	Ghana	7 districts	Rural	2009	Facility	Cross-sectional	Mixed	Midwife/nurse/CHW	134		√	√	√
Stangeland 2011 [45]	East Africa	Uganda	<1 district	Rural	2009	Population	Cross-sectional	Mixed	TBA	28	√		√	
Tawfik 2006 [17]	Asia	Cambodia	2 districts	Urban	2004	Population	Cross-sectional	Mixed	Pharm/DV	70	√	√	√	
Umar 2011 [38]	West Africa	Nigeria	1 state	Urban	—	Facility	Cross-sectional	Quantitative	FHW	25	√	√	√	√
Wylie 2010 [15]	Asia	India	2 states	Rural/urban	2006–2008	Facility	Cross-sectional	Quantitative	PW+HP†	280	√		√	√

§ Health provider practices inferred from medical file/ANC card.

† Health provider practices observed.

CHW, community health worker; DV, drug vendor/drug store; FHW, female health worker; HP, health provider; MA, medical assistant; MD, medical doctor; pharm, pharmacist (trained); PSI, Population Services International Research and Metrics; PW, exit interviews with pregnant women.

**Table 3:** Characteristics of studies reporting outcomes for both pregnant women and health providers (six studies).

Study Description										*N*		Primary Outcome (PW/HP)*						
Study	Region	Country	Scale	Urban/Rural	Study Year	Target Population	Study Design	Data Type	Sample	PW	HP	1	2	3	4		5	6
															D	T		
Kamuhabwa 2011 [35]	East Africa	Tanzania	<1 district	Urban	2009–2010	Facility	Cross-sectional	Quantitative	PW+DV	200	200		√	√		√	√	√
Kwansa-Bentum 2011 [30]	West Africa	Ghana	1 district	Rural/urban	2010	Population	Cross-sectional	Quantitative	PW+MD/nurse/pharm	959	126	√	√			√	√	
Manirakiza 2011 [36]	Central Africa	CAR	<1 district	Urban	2009	Facility	Cross-sectional	Quantitative	PW+HP§	565			√		√	√	√	
Mbonye 2010 [52]	East Africa	Uganda	1 district	Rural/urban	—	Population	Cross-sectional	Quantitative	PW+TBA/DV/CHW	2,785	51		√				√	
Obieche 2013 [28]	West Africa	Nigeria	<1 district	Urban	2011	Facility	Cross-sectional	Quantitative	PPW+HP§	428		√			√	√		
Pell 2013 [21]	East, west, southern Africa	Kenya, Ghana, Malawi	4 districts	Rural/urban	2009–2011	Population	Anthropological	Qualitative	PW+HP	390	137		√				√	

*Primary outcomes for both pregnant women and health providers: (1) treatment-seeking practices, (2) barriers to treatment seeking, (3) determinants of treatment seeking, (4) knowledge and practices for case management of malaria (diagnosis/treatment), (5) barriers to case management, and (6) determinants of case management.

§ Health provider practices inferred from medical file/ANC card.

CHW, community health worker; D, diagnostics; DV, drug vendor/drug store; HP, health provider; MD, medical doctor; pharm, pharmacist (trained); PPW, postpartum women; PW, pregnant women; T, treatment.

Quality of about half (14/27) of the quantitative studies was assessed to be moderate-high (scored 6–8/10), with ten low-moderate-quality studies (4–5/10) and three high-quality studies (9–10/10) (Table S2). The quality criterion least often met among these studies was the use of multivariate analysis. The four qualitative studies were assessed as moderate-high quality (4–7/8), with only one of the studies reporting saturation of themes (Table S3). All six mixed methods studies were assessed as high quality (9–10/11), though only one study reported use of multivariate analysis (Table S4). Data on frequencies of practices, barriers/facilitators, and determinants of access among women were extracted from 13, 15, and four studies, respectively, and of policy adherence among healthcare providers, from 24, 22, and ten studies, respectively (Table 4).

**Table 4:** Data extracted for frequencies, barriers, and determinants by survey type.

Study	Pregnant Women†			Healthcare Providers≠		
	Frequencies	Barriers	Determinants	Frequencies	Barriers	Determinants
**Facility-based studies**						
Bin Ghouth 2013 [18]				√	√	√
Kiningu 2013 [41]				√	√	√
Luz 2013 [19]				√		√
Luz 2013 [20]				√	√	
Mbonye 2013 [34]				√		
Obieche 2013 [28]	√			√		
Onwujekwe 2013 [29]	√			√		
Harrison 2012 [43]				√	√	√
Minyaliwa 2012 [51]				√	√	
Okoro 2012 [40]				√	√	
Onwujekwe 2012 [39]				√	√	√
Kamuhabwa 2011 [35]	√	√	√	√	√	√
Manirakiza 2011 [36]		√		√	√	
Smith Paintain 2011 [47]				√	√	√
Umar 2011 [38]				√	√	√
Karunamoorthi 2010 [32]	√	√				
Launiala 2010 [22]		√				
Maiga 2010 [12]	√	√				
Sabin 2010 [14]	√	√				
Smith Paintain 2010 [23]		√				
Wylie 2010 [15]				√	√	√
Enato 2009 [27]	√	√				
Omo-Aghoja 2008 [37]				√	√	√
PSI 2007 [16]				√	√	
**Population-based studies**						
Pell 2013 [21]		√			√	
Enato 2012 [44]				√	√	
Mbachu 2012 [24]	√	√	√			
Henry 2012 [26]	√		√			
Kalilani-Phiri 2011 [42]				√	√	
Kwansa-Bentum 2011 [30]	√	√		√	√	
Okonta 2011 [46]				√	√	
Sangaré 2011 [25]	√	√	√			
Stangeland 2011 [45]				√	√	
Mbonye 2010 [52]		√			√	
Adam 2008 [31]	√	√				
Sam-Wobo 2008 [33]	√	√				
Tawfik 2006 [17]				√	√	
Summary total	13	15	4	24	22	10

† Pregnant women: for frequency data, see Tables 5 and 6; barrier data, Table 7; determinant data, Table 8.

≠ Healthcare provider: for frequency data, see Tables 9 and 10; barrier data, Table 11; determinant data, Table 12.

PSI, Population Services International Research and Metrics.

### Pregnant Women Perspectives

The 19 studies that contributed data on the treatment-seeking practices of pregnant women were undertaken in ten countries across Africa (seven studies in east Africa, eight in west Africa, one in southern Africa, one in central Africa, and one with sites in east, west, and southern Africa) and in one country in Asia (Tables 1 and 3).

#### Description and frequency of practices among pregnant women

The proportion of women reporting at least one episode of malaria during their current or recent pregnancy ranged from 25% to 75% of respondents in three population-based [24]–[26] and three facility-based [14],[27],[28] studies in Africa and Asia, with between 30% and 46% of women reporting two or more episodes in Africa [25],[27],[28] (Tables 5 and 6). Of one population-based [25] and three facility-based [14],[28],[29] studies, a high proportion (>85%) of women with a reported episode of malaria during pregnancy sought some form of treatment.

**Table 5:** Symptoms and number of episodes of malaria in pregnancy, and percentage who sought treatment by source, reported by pregnant women: population-based studies.

Region	Country	Study	Scale	*N*	Reported an Episode of Malaria in Pregnancy	Number of Episodes Reported per Pregnancy	Percentage of Women Who Sought Treatment	Source of Treatment				
								HCF/ANC	Private Clinic	Retail Sector/Pharmacy	Self-Medicate	Traditional
**West and Central Africa**	Ghana	Kwansa-Bentum 2011 [30]	1 district	959	NR	NR	NR	25.4%		28.8%§		5.4%†
	Nigeria	Mbachu 2012 [24]	1 district	898	25.3% (fever)	NR	NR	42.3%				
	Nigeria	Sam-Wobo 2008 [33]	1 district	1,400	NR	65.0% of PW had 3–4 episodes of malaria that year (not current pregnancy)	NR					68.0%∞
**East and Southern Africa**	Sudan	Adam 2008 [31]	1 district	168	NR	NR	NR	81.5%∧			9.5%	
	Uganda	Henry 2012 [26]	1 district	769	49.0% in past 2 mo	NR	NR	86.0%¢	10.0%	4.0%		
	Uganda	Sangaré 2011 [25]	<1 district	500	66.8%	37.0% had 2+ episodes of malaria in pregnancy	94% of reported episodes					

§ Multiple response answers.

† Herbs; <1% sought prayers, baths, water, and/or sleep.

∞ Specified as herbs.

∧ 8.9% specifically sought a midwife.

¢ IDP camp setting.

HCF, healthcare facility; NR, not reported by study authors; PW, pregnant women.

**Table 6:** Symptoms and number of episodes of malaria in pregnancy, and percentage who sought treatment by source, reported by pregnant women: facility-based studies.

Region	Country	Study	Scale	*N*	Percentage of Women Who Reported an Episode of Malaria in Pregnancy	Number of Episodes Reported per Pregnancy	Percentage of Women Who Sought Treatment	Source of Treatment			
								HCF/ANC	Retail Sector/Pharmacy	Self-Medicate	Traditional
**West and Central Africa**	Mali	Maiga 2010 [12]	<1 district	210	NR	NR	NR	31.4%		40.0%	27.6%
	Nigeria	Obieche 2013 [28]	<1 district	428	69.4%	30% reported >1 episode	84.6% of reported episodes	77.4%	10.7%	12.0%	
	Nigeria	Onwujekwe 2013 [29]	<1 district	647	NR	NR	Women attending public facilities, 95.3%	89.1%	5.7%	5.2%	
							Women attending private facilities, 98.6%	92.0%	2.3%	6.0%	
	Nigeria	Enato 2009 [27]	1 state	630	64.1%	1 episode, 53.7%; 2, 27.3%; 3, 6.3%; and 4+, 12.7%	NR	78.0%		22.0%	
**East and Southern Africa**	Ethiopia	Karunamoorthi 2010 [32]	<1 district	225	NR	NR	NR	88.1%		7.4%	4.5%α
	Malawi	Launiala 2010 [22]	<1 district	34	NR	NR	NR	Second choice	Majority		
**Asia**	India	Sabin 2010 [14]	1 state	73*	75.0%	NR	85.0% of reported episodes	63.0%		20.8%	16.7%¥

α Specified as TBA.

*Pregnant women and recently delivered women.

¥ Specified as traditional remedies.

HCF, healthcare facility; NR, not reported by authors.

Pregnant women in three population-based [26],[30],[31] and seven facility-based [12],[14],[22],[27]–[29],[32] studies in Africa reported self-medication or treatment at a pharmacy/drug store at the onset of fever (range 5%–40%), and attended a health facility only if their fever did not respond to this treatment [12],[30] (Tables 5 and 6). In southern Ghana, women seeking treatment at a pharmacy or drug vendor without a clinic prescription reported that the antimalarials were selected by either the shop attendant (21% and 26% in rural and urban areas, respectively) or themselves (8% and 10%, respectively) [30]. Use of local herbs was a first resort among pregnant women in a population-based study in Nigeria [33]. Pregnant women in urban settings were likely to seek care from antenatal care (ANC) or health facilities as a first resort, as observed in three population-based studies in Nigeria (42%) [24], Sudan (82%) [31], and internally displaced person (IDP) camps in Uganda (86%) [26], and five facility-based studies (range 63%–92%) in Ethiopia [32], Nigeria [27]–[29], and India [14].

**Figure 3:**
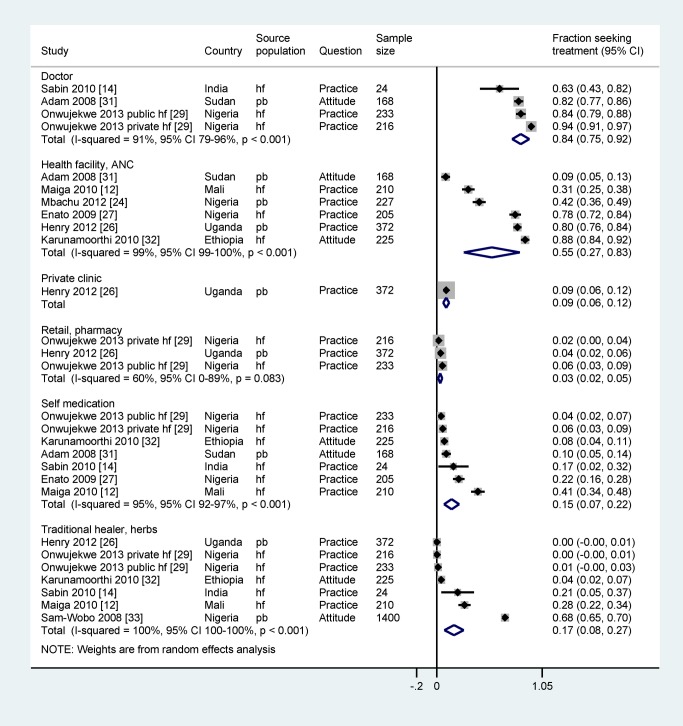
Prevalence of source of malaria treatment during pregnancy assessed in 18 studies with quantitative data. hf, health facility–based survey; pb, population-based survey.

Data on sources of treatment extracted from nine studies showed high heterogeneity across studies (*I* ^2^ ranging from 60% to 99%) (Figure 3), and all but one study [12] were of moderate to high quality. Site of enrolment (health facility– versus population-based), country (Nigeria versus other countries), and type of question (practice versus attitude) had no effect within each category (Table S5). Compared to urban women, rural women were more likely to make use of a traditional healer or herbs (2%, 95% CI 0%–7%, three studies, versus 21%, 95% CI 6%–52%, four studies, respectively, *p* = 0.008), whereas urban women made more use of health facilities (84%, 95% CI 71%–91%, two studies, versus 38%, 95% CI 14%–70%, four studies, *p* = 0.006).

Only six of the 14 studies among pregnant women with treatment-seeking practice as a primary outcome included quantitative data on use of ACTs, and of these, only four stratified use by trimester. In a household survey in Uganda, among first trimester episodes, quinine was used in only 6% of first trimester cases, with >80% of episodes treated with drugs not recommended for use in the first trimester either because they are contraindicated (sulphadoxine-pyrimethamine [SP] and artemether-lumefantrine [AL]) or because of high-grade drug resistance (chloroquine [CQ]) [25]. Only 30% of second and third trimester cases adhered to national guidelines (AL or quinine) [25]. Another study conducted in 2011 in Uganda reported appropriate treatment in only 36% of febrile cases, defined as parasite-positive pregnant women given AL (Coartem) and parasite-negative women given no antimalarial drug [34]. Of pregnant women interviewed at a public hospital in Tanzania, 31% had used AL for an episode of malaria in the index pregnancy, 27% SP, 23% quinine, 16% sulphalene-pyrimethamine, and 3% amodiaquine (AQ) [35]. The majority (82%) of women said they were asked about gestational age before being given AL by drug dispensers; however, only 17% of pregnant women were aware that AL should not be taken in the first trimester, and only 22% knew that quinine was recommended. In a population-based study in Ghana, drug sellers said some women requested artemisinin combinations for treatment in the first trimester [30].

A household survey in southeast Nigeria found that 42% of pregnant women who had a fever within the last month had visited a health facility, of which 46% were treated with ACTs, 34% with SP, and 4% with artemisinin monotherapy; however, trimester was not specified [24]. In an earlier population-based study in Nigeria, women reported a high preference for SP for case management in the second and third trimesters of pregnancy, whereas the national treatment policy in second and third trimesters was to use AL; the study was, however, done only a year after the new policy was introduced [33]. A more recent facility-based study in a teaching hospital reported that quinine was used in only 12% of first trimester episodes, with artemisinin-containing compounds, SP, and CQ used in 35%, 39%, and 14% of cases, respectively [28]. In a comparative study of treatment practices of second and third trimester episodes in public and private health facilities, quinine was used in 4% of episodes in both types of facility, with artemisinin monotherapy constituting the most frequently prescribed drug (36%–39%) [29].

#### Barriers to care seeking for malaria among pregnant women

The factors affecting treatment seeking for malaria most frequently cited in the content analysis related to the following: women's knowledge and perceptions of risk, perceptions and experience of drug safety, cost, and perceptions and experience of healthcare provider and health facility factors (Table 7). Women in one facility-based study [14] perceived malaria during pregnancy as not especially dangerous, and the first response in two population-based [30],[31] and four facility-based [12],[14],[22],[32] studies was to rely on self-medication or herbal treatments, and to seek medical advice only if the illness did not improve. Over 50% of women in a facility-based study reported delaying >2 d after first noticing symptoms before seeking care [27]. The choice of treatment was influenced by women's perceptions of the safety of drugs used during pregnancy, as reported by three population-based studies [30],[31],[33] and one facility-based study [35]. Fear and/or prior experience of side effects to drugs also influenced treatment choices and adherence, as reported by one population-based [21] and three facility-based [14],[23],[35] studies. In northern Ghana, pregnant women identified contradictions between messages provided in health facilities and their own experiences of malaria [21].

**Table 7:** Content analysis of factors that affect treatment seeking for malaria among pregnant women.

Barriers and Facilitators to Treatment Seeking for Pregnant Women			Level							
			Individual		Social/Cultural/Household		Environmental		Health System	
			Quan	Qual	Quan	Qual	Quan	Qual	Quan	Qual
**Barriers**	Knowledge	Low perceived danger of malaria in pregnancy	1	2						
		Low knowledge of treatment measures	5	3						
		Reliance on self-medication/herbal treatments	5	4						
		Pregnant women considered less of a priority or vulnerable			0	1				
	Safety	Perception of safety of drugs during pregnancy	3	1						
		Fear of side effects	1	3						
		Experience of side effects	0	2						
	Cost	Cost of treatment			4	3				
		Travel costs to health care facility			0	3				
		User fees							2	2
		Husband controls finances			0	1				
	Health facility	Drug stock-outs							1	1
		Lack of trust in provider/confusion about healthcare provider advice for treatment	0	1						
		Lack of adequate care at health care facility							0	1
**Facilitators**	Knowledge	Concern for status of pregnancy	0	2						
		Awareness of treatment options	1	2						
		Trust in health care facility/medication	0	2						
	Safety	Belief that drugs are safe to use	2	1						
		Treatment considered as effective	4	3						
		Very few or no side effects	1	0						

Numbers indicate the number of studies included in this review that report each factor.

qual, qualitative; quan, quantitative.

The high cost of treatment prevented pregnant women from using the formal health sector in rural population-based surveys in Ghana [21],[30], Kenya [21], and Nigeria [24]. Poverty was said to be why women resorted to herbal remedies in Kenya and Ghana, to avoid costs of both transport and medical care [21]. Other barriers cited were user fees at formal health services [33] or the cost of treatment in urban areas in population-based surveys in Ghana [30] and facility-based studies in the Central African Republic (CAR) [36] and India [14]. Lack of adequate care at health facilities [23] was an additional deterrent to using the health facilities. Women in one study reported that they did not understand the instructions given by dispensers regarding (AL) dosage and duration of use [35]. On the other hand, women who were concerned for their pregnancy status, who were aware of the treatment options [22],[23],[30] and considered the drugs safe and effective [14],[25],[30],[35], and who trusted the health facility staff [21],[23] were more likely to seek treatment at health facilities. Women in Ghana and Kenya generally valued diagnostic tests for malaria (and other diseases) and associated testing with more effective treatment [21].

#### Determinants of care seeking for malaria among pregnant women

The range of determinants of treatment seeking among pregnant women explored across the included studies included education, prior experience of miscarriage, and ANC use. The key findings are highlighted in Table 8; insufficient data and lack of consistency in the indicators used prevented us from performing a meta-analysis of pooled data. A higher level of education was associated with correct knowledge of AL use in pregnancy in Tanzania [35]. Prior use of ANC services and previous experience of miscarriage were associated with increased treatment seeking for malaria in IDP camps in Uganda [26].

**Table 8:** Data on the determinants of treatment-seeking behaviours for malaria in pregnancy by pregnant women.

Determinant	Study	Country	Scale	*N*	Details
Age	Kamuhabwa 2011 [35]	Tanzania	<1 district	200	Age is not associated with knowledge of AL use in pregnancy
	Henry 2012 [26]	Uganda	1 district	769	Age is not associated with increased treatment seeking
Education	Kamuhabwa 2011 [35]	Tanzania	<1 district	200	A higher level of education in women was associated with correct knowledge of AL use in pregnancy (*p*<0.001)
	Henry 2012 [26]	Uganda	1 district	769	Women's level of education was not associated with increased treatment seeking
Marital status	Henry 2012 [26]	Uganda	1 district	769	Marital status was not associated to increased treatment seeking
Parity/gravidity	Kamuhabwa 2011 [35]	Tanzania	<1 district	200	Parity/gravidity was not associated with knowledge of AL use in pregnancy
	Henry 2012 [26]	Uganda	1 district	769	Gravidity was not associated with increased treatment seeking
	Sangaré 2011 [25]	Uganda	1 district	500	There was no difference between multiparous and primiparous women in their use of the recommended dosage of treatment
Gestational age	Kamuhabwa 2011 [35]	Tanzania	1 district	200	Age of gestation was not associated with knowledge of AL usage in pregnancy
	Henry 2012 [26]	Uganda	1 district	769	Age of gestation was not associated with increased treatment seeking
Experience of miscarriage	Henry 2012 [26]	Uganda	1 district	769	Prior experience of miscarriage was associated with increased treatment seeking (*p* = 0.049)
Prior use of ANC	Henry 2012 [26]	Uganda	1 district	769	Prior use of ANC services by women was associated with increased treatment seeking (*p* = 0.029)
SES	Mbachu 2012 [24]	Nigeria	1 district	898	SES of women was not associated with the utilisation of different antimalarials by pregnant women

All effects measured using the Chi-squared test.

SES, socio-economic status.

### Healthcare Provider Perspectives

The 24 studies that contributed data on the diagnosis and treatment practices of healthcare providers were undertaken in ten countries, involving a range of cadres, including medical doctors and nurses, pharmacists, drug vendors, traditional birth attendants (TBAs), and community health workers (CHWs) (Tables 2 and 3).

#### Description and frequency of diagnostic practices

Malaria diagnosis in pregnancy by public healthcare providers in the studies conducted in Africa was predominantly performed on the basis of clinical symptoms, as reported by one population-based study in Ghana [30] and five facility-based studies in CAR [36] and Nigeria [37]–[40]. The exceptions to this were microscopy use by private sector providers in Nigeria [39] and by a provincial hospital in Kenya [41] (Table 9). One population-based study in Malawi [42] and three facility-based studies in Nigeria [37],[38],[43] reported a combination of clinical and parasitological diagnosis by microscopy. Providers at the community level in three population-based studies, including private providers in Cambodia [17] and TBAs in Africa [44],[45], relied exclusively on clinical symptoms unless women could produce prescriptions issued from clinics. Globally, few studies reported healthcare providers using RDTs. In Africa, reports of RDT use have been relatively recent (2011 in Malawi [42] and 2012 in Nigeria [39]), compared to in Asia (2007 in Cambodia [16]), and only a fraction of providers reported using RDTs (range 22%–34%) [16],[39],[42]. In a population-based survey of medical doctors and pharmacists in Malawi, availability of tests, patient symptoms, and cost were the main factors affecting choice of diagnostic test [42]. In an observational study of ANC visits in eastern India, blood tests were typically obtained if a patient complained of fever, though enquiries into presence of fever in patients were made in only a minority of patients [15].

**Table 9:** Healthcare provider practices: diagnosis.

Region	Country	Study	Policy Reference	Policy Details: Diagnosis		Scale	Reported Provider Practice					
							Type of Healthcare Provider	*N*	Diagnosis			
									Clinical		Parasitological	
				Clinical	Laboratory				Clinical Diagnosis	Clinical Symptoms	Microscopy	RDT
**West and Central Africa**	Nigeria	Obieche 2013 [28]	National Antimalarial Treatment Guidelines and Policy, 2005		Microscopy/RDT	<1 district	Medical records and interviews with PW				8.6%	
	Nigeria	Harrison 2012 [43]	National Antimalarial Treatment Guidelines and Policy, 2005	Fever, pallor	Microscopy/RDT	1 district	MD	123	85.4%∧		85.4%∧	
	Nigeria	Okoro 2012 [40]	National Antimalarial Treatment Guidelines and Policy, 2005	Fever, pallor	Microscopy/RDT	<1 district	HP	311	80.0%		20.0%	
	Nigeria	Onwujekwe 2012 [39]	National Antimalarial Treatment Guidelines and Policy, 2005	Fever, pallor	Microscopy/RDT	<1 district	Public: MD/nurse/pharm	32	78.1%§		65.6%	43.8%
							Private: MD/nurse/pharm	20	47.4%§		68.4%	15.8%
	Nigeria	Enato 2012 [44]	National Antimalarial Treatment Guidelines and Policy, 2005	Fever, pallor	Microscopy/RDT	<1 district	TBA	8	100.0%	Fever, colour of urine, dizziness, blood pressure, weakness, and appetite		
	Nigeria	Umar 2011 [38]	WHO guidelines 2010	Fever, pallor, anaemia	Microscopy/RDT	1 state	HP	25	69.3%€		87.5%α	
	Nigeria	Omo-Aghoja 2008 [37]	National Antimalarial Treatment Guidelines and Policy, 2005	Fever, pallor	Microscopy/RDT	National	MD	84	62.0%†		26.0%†	
**East and Southern Africa**	Kenya	Kiningu 2013 [41]	National Malaria Guidelines, 2010		Microscopy/RDT	<1 district	Medical records	37	5.5%		91.9%	
	Malawi	Kailani-Phiri 2011 [42]	Malawi ACT guidelines, 2008			National	MD/pharm	92	84.1%	Used symptoms in addition to lab tests§ ^,^ µ	73.1%	25.6%µ/1.20%¢
	Uganda	Stangeland 2011 [45]	National Malaria Treatment Guidelines, 2005¥	History and physical exam	Microscopy/RDT	<1 district	TBA	28	100.0%	75% fever, 75% shivers, 39% headache, 29% vomiting, 25% pale eyes, 25% no appetite, 25% weakness, 21% abdominal pains§		
**Asia**	Cambodia	PSI 2007 [16]	NA			National	MD/MA/pharm/nurse/midwife/DV	750				89.0%∞
	Cambodia	Tawfik 2006 [17]	WHO/Cambodia National Treatment Guidelines 2002			2 districts	Pharm/DV/CHW	70	3.0%		94.0%±	
	India	Wylie 2010 [15]	Indian National Drug Policy, 2007	Fever, chills, headache, joint pain	Microscopy/RDT	2 states: region A	MD	120	20.0%/40.8%≠	Fever/signs of anaemia§	14.2%	
						2 states: region B	MD	160	48.1%/75.0%≠	Fever/signs of anaemia§	37.5%	

∧ Equal numbers used clinical and laboratory tests.

§ Multiple response answers.

€ Lower cadre providers (senior and junior community health extension workers and pharmacy technicians).

α Higher cadre providers (doctors, nurses, and community health officers).

† Used clinical and lab-based tests: 62% sometimes and 26% always.

µ 25.6% used both RDT and microscopy.

¢ Used RDTs only.

¥ Policy document identified by review authors.

∞ Frequency of RDT use: always 9.2%, most of time 33.6%, sometimes 22.7%, rarely 26.1%, never 8.4%.

± Microscopy or RDT; 3% used clinical and lab.

≠ Healthcare provider asks about presence of fever/assesses for signs and symptoms of anaemia.

CHW, community health worker; DV, drug vendor/shop; HP, healthcare provider; MA, medical assistant; MD, medical doctor; NA, not reported by study authors; pharm, pharmacist (trained); PSI, Population Services International Research and Metrics; PW, pregnant women.

#### Description and frequency of treatment knowledge and practices

In west and central Africa, 11 studies on health providers were conducted in Nigeria (eight studies), Ghana (two), and CAR (one), where the national antimalarial treatment guidelines stipulate quinine for treatment of uncomplicated malaria in the first trimester and an ACT in the second and third trimesters [30],[36],[39] (Table 10). Only two of the eight studies in Nigeria [28],[37]–[40],[43],[44],[46] reported a relatively high proportion of providers adhering to treatment policy. Onwujekwe et al. found that more doctors, pharmacists, and nurses providing ANC services in public than private sector hospitals adhered to the national policy of prescribing ACTs in the second and third trimesters (69% versus 5%); private hospitals predominantly prescribed SP (70%) [39]. More public than private sector providers prescribed quinine in the first trimester (35% versus 15%); private sector providers predominantly prescribed SP (65%). Okonta found that whilst 56% of doctors had prescribed quinine during the first trimester, the fear of quinine causing miscarriage was a significant consideration, with all but one physician prescribing quinine at lower than the recommended dose, and showing a preference for CQ [46]. Similarly, Okoro and Nwambu found very low prescription of quinine in the first trimester (2.5%), and ACTs constituted 51% and 29% of antimalarial drugs prescribed in the second and third trimesters, respectively [40]. Two studies did not stratify treatment drug by trimester [37],[43]. Up until 2012, SP continued to be used widely for case management of clinical malaria in pregnancy in Ghana [30],[47] and Nigeria [28],[37]–[40],[43], as well as CQ, despite its known resistance [37],[39],[43], and artemisinin monotherapies [37],[43],[46].

**Table 10:** Healthcare provider practices: antimalarials prescribed.

Region	Country	Study	Policy Reference	Policy Details: Treatment		Scale	Reported Provider Practice				
							Healthcare Provider and Method of Data Collection	*N*	Type of Drug Prescribed by Trimester of Pregnancy		
				First Trimester	Second/Third Trimester				First Trimester	Second/Third Trimester	Trimester Not Specified
**Middle East**	Yemen	Bin Ghouth 2013 [18]	WHO guidelines 2010	NR	NR	11 districts	Clinicians/pharm/drug store employee; structured questionnaire	86			Pre-intervention: AS 47.0%, CQ 19.0%, QN 17.0%; post-intervention: AS 19.0%, CQ 22.0%, QN 60.0%
**West and Central Africa**	CAR	Manirakiza 2011 [36]	WHO guidelines 2006	QN	ACT	<1 district	ANC staff≠; review of ANC cards	565	QN 68.6%, ACT 17.1%, AS 11.4%	2nd trimester: QN 55.5%, ACT 34.2%, AS 18.8%	
	Ghana	Kwansa-Bentum 2011 [30]	Ghana Health Service, 2009	QN	AS-AQ/AL/DHA-PPQ	1 district	HP; interviews	88	QN 45.0%, SP 10.0%,	AS-AQ 45.0%, QN 20.0%, SP 20.0%, AL 5.0%	
							DV; interviews	38	SP 10.0%, QN 5.0%, AL 5.0%, DHA-PPQ 5.0%, AS-AQ 2.0%, AS 1.0%	DHA-PPQ 10.0%, SP 12.0%, QN 5.0%, AL 5.0%, AS-AQ 3.0%	
	Ghana	Smith Paintain 2011 [47]	Ghana Health Service, 2009	QN	AS-AQ	7 districts	Midwife/nurse/CHW	134	Knowledge: QN 50.8%, AS-AQ 20.2%, AS 14.2%, SP 7.5%	Knowledge: AS-AQ 78.4%	
	Nigeria	Obieche 2013 [28]	National Antimalarial Treatment Guidelines and Policy, 2005	QN	QN	<1 district	Postpartum women; interview/medical record check	428	SP 38.8%, CQ 14.3%, QN 12.2%, AL 24.5%, AS 8.2%, AS inj. 2%	AL 49.6%, SP 24%, AS 13.4%, AS inj. 2.4%, CQ 4.4%, QN <1%, AS-SP 1.9%, AS-AQ 1%, AQ 2.4%	
	Nigeria	Harrison 2012 [43]	National Antimalarial Treatment Guidelines and Policy, 2005	QN	AL	1 district	MD; self-administered questionnaire	123			CQ 22.8%, SP 21.1%, camoquine 10.6%, AL 4.1%, QN, 3.3%, AS 1.6%, camoquine/SP 1.6%
	Nigeria	Okonta 2011 [46]	National Antimalarial Treatment Guidelines and Policy, 2005	QN	AL	National	MD; self-administered questionnaire	102	CQ 40.2%, QN 19.6%, AQ 14.7%, SP 8.8%, AS 6.9%		
	Nigeria	Okoro 2012 [40]	National Antimalarial Treatment Guidelines and Policy, 2005	QN	AL	<1 district	HP; medical card reviews	311	SP 12.5%, QN 2.5%, ACT 2.5%, CQ 1.25%	ACT 80.0%, QN 1.3%	
	Nigeria	Onwujekwe 2012 [39]	National Antimalarial Treatment Guidelines and Policy, 2005	QN	AL	<1 district	Public sector: MD/nurse/pharm; self-administered questionnaire	32	QN 34.5%, CQ 21.9%, SP 12.5		ACT 68.8%, QN 50.0%
							Private sector: MD/nurse/pharm; Self-administered questionnaire	20	SP 65.0%, QN 15.0%, CQ 15.0%		SP 70.0%, QN 25.0%, ACT 25.0%
	Nigeria	Enato 2012 [44]	National Antimalarial Treatment Guidelines and Policy, 2005	QN	AL	<1 district	TBA	8			Some referred to CQ use
	Nigeria	Umar 2011 [38]	WHO guidelines 2010	QN	ACT	1 state	HP; self-administered questionnaire	25	SP 68.0%, CQ16.0%, AL 8.0%, SP+CQ 4.0%, QN+CQ 4.0%		
	Nigeria	Omo-Aghoja 2008 [37]	National Antimalarial Treatment Guidelines and Policy, 2005	QN	AL	National	MD; self-administered questionnaire	84			CQ 73.0%, SP 10.0%, AS 11.0%, QN 3.0%, AQ 1.0%
**East and Southern Africa**	Kenya	Kiningu 2013 [41]	National Malaria Guidelines, 2010	Mild/moderate QN or AL	AL	<1 district	Medical files	37			QN IV 73.0%, AL 2.7%, QN 2.7%
	Malawi	Minyaliwa 2012 [51]	Malawi ACT guidelines, 2008	QN	AL/AS-AQ	1 district	Pharm; interviews	22	QN 90.9%	ACT 90.9%	
	Tanzania	Kamuhabwa 2011 [35]	WHO guidelines 2006	QN	AL	<1 district	Drug dispenser (all)	200	AL 32.8%		
							Pharm/mystery client	60	QN 55%, SP 19.4%, DHA-PPQ 17.6%		
							Pharm Ass/mystery client	34	QN 16.6%, SP 22.6%, DHA-PPQ 23.5%, AQ 25%, sulphalene-pyrimethamine 33.3%		
							Nurse Ass/mystery client	71	QN 22.2%, SP 32.5%, DHA-PPQ 23.5%, sulphalene-pyrimethamine 16.6%		
							DV/mystery client	35	QN 5.5%, SP 25.9%, DHA-PPQ 33.3%, AQ 75%, sulphalene-pyrimethamine 50%		
**Asia**	Cambodia	Tawfik 2006 [17]	WHO/Cambodia National Treatment Guidelines 2002	Pf: QN; Pv/Pm: CQ	Pf: ART/MQ; Pv/Pm: CQ	2 districts	Pharm/DV/CHW; client interviews	70	QN 14.8%		
**South America**	Brazil	Luz 2013 [19]	Brazil malaria treatment guidelines, 2001/2008	Pv: CQ; Pf: QN or QN/CN	AL or MQ	>1 district	Medical records	262			Pv: CQ 91%, CQ combo 2.7%, MQ 2.7%, QN 2.1%, AL 1.6%;Pf: MQ 37.8%, QN+CN 18.9%, QN 13.5%, CQ 8.2%, MQ combo 2.7%, AL 16.2%, ART 1.4%, CN 1.4%

≠ Data obtained from ANC cards.

ART, artemether monotherapy; Ass, assistant; CHW, community health worker; CN, clyndamicine; DHA-PPQ, dihydroartemisinin-piperaquine combination; DV, drug vendor/shop; HP, healthcare provider; inj., injected; MD, medical doctor; MQ, mefloquine; NA, not reported by authors; Pf, *P. falciparum*; pharm, pharmacist; Pm, *P. malariae*; Pv, *P. vivax*; QN, quinine.

In Ghana, Kwansa-Bentum et al. found clinicians prescribing quinine for malaria treatment in the first trimester of pregnancy, per policy, but also SP, and predominantly ACTs were prescribed in the second and third trimesters, though SP and quinine were also prescribed [30]. In the same study, drug sellers reported pregnant women requesting, in order of preference, SP, ACTs, or quinine, with no difference by trimester. Smith Paintain et al. found that few ANC providers (20%) demonstrated good knowledge of the dosing regimen for treatment in the first trimester, though knowledge that AS-AQ should be prescribed in subsequent trimesters was better (42%), with preference for AS-AQ over quinine due to the side effects and long regimen duration (7 d) of quinine [47]. In CAR, 29% of ANC cards of women who had delivered in a maternity unit contained at least one antimalarial prescription, of which 57% were for quinine, 27% for ACTs, and 14% for artemisinin monotherapies; 11% and 13% of ACTs and artemisinin monotherapies, respectively, were for treatment in the first trimester [36].

In east and southern Africa, six studies were conducted in Kenya (one study), Malawi (two), Tanzania (one), and Uganda (two), where, again, the national malaria treatment policies recommend quinine in the first trimester, with an ACT (AS-AQ or AL) for the second/third trimester [48]–[50]. In Kenya, while 83% of staff at a provincial hospital stated that they used the national guidelines, guidelines were available at only 25% of points of use, and 73% of pregnant patients received parenteral quinine [41]. In Malawi, Kalilani-Phiri et al. found that only 40% of medical doctors and pharmacists knew the treatment guidelines for uncomplicated malaria in pregnant women, compared to 83% for severe malaria [42]. In contrast, Minyaliwa et al. reported that a high proportion (91%) of providers were cognisant of the appropriate drugs to use in each trimester, though specific drug names were not reported [51]. A study of dispensing practices among private pharmacies in urban Tanzania found low knowledge of appropriate antimalarial drugs, with 33% of providers willing to dispense AL for use in the first trimester and 36% indicating it could not be used in pregnancy. Nevertheless, 82% of women reported that they were asked about gestational age before they were given AL. Pharmacists and nurse assistants had better AL knowledge than pharmaceutical technicians and sales persons [35]. In Uganda, Mbonye and Magnussen found that 38% of pregnant women with reported fever but negative blood smears received an antimalarial (drug not specified) in addition to IPTp [52].

The remaining studies were conducted in Cambodia (one study) and Brazil (two studies). In Cambodia, only 4/27 pregnant women surveyed received the recommended drug, quinine, in the first trimester, and knowledge amongst private providers about first line treatment for malaria in pregnancy was poor [17]. In Brazil, while 93% of patients received the recommended first line therapy for *Plasmodium vivax* malaria, only 45% of patients received the recommended first line therapy for *P. falciparum* malaria, with 7% and 18% of prescriptions, respectively, not sanctioned by national guidelines [20].

#### Meta-analysis of adherence to treatment policy

**Figure 4:**
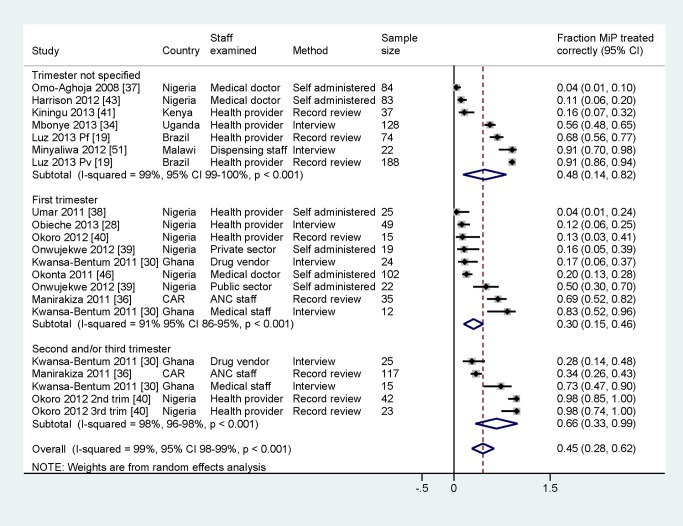
Prevalence of adherence to treatment policy for malaria in pregnancy assessed in 15 studies with quantitative data. Pf, *P. falciparum*; Pv, *P. vivax*; self-administered, self-administered questionnaire; MiP, malaria in pregnancy.

Frequency data on adherence among healthcare providers to treatment policy by trimester, extracted from 12 studies, showed wide heterogeneity (overall *I* ^2^ 98.6%) (Figure 4), and all but one study [51] were of moderate to high quality. There was lower adherence to treatment policy in the first trimester (28%, 95% CI 14%–47%, nine entries from seven studies) than in the other trimesters (72%, 95% CI 39%–91%, five entries from three studies), and this difference was significant in the sub-group analysis (*p* = 0.02) (Table S6). Studies describing practices among doctors (three entries from Nigeria, 269 doctors in total as defined by the local researchers, all self-administered questionnaires) found that these healthcare providers were significantly less likely to prescribe correctly (11%, 95% CI 4%–23%) than healthcare providers in studies describing practices among other staff or mixed cadres (18 entries, 52%, 95% CI 35%–67%, *p*<0.001). Studies conducted by self-administered questionnaires showed a significantly lower proportion of adherence to treatment policy (14%, 95% CI 7%–28%, six entries) than studies using interviews (50%, 95% CI 27%–73%, seven entries) or record reviews (66%, 95% CI 39%–86%, eight entries, *p* = 0.001), and studies describing practices in Nigeria were significantly less likely to report correct treatment (25%, 95% CI 12%–46%, ten entries from eight studies) than studies in all other countries combined (58%, 95% CI 40%–75%, 11 entries from eight countries, *p* = 0.018).

#### Barriers to effective case management practices for malaria in pregnancy among healthcare providers

Factors affecting diagnostic and case management practices occurred at all levels of the health system and across all of the health system building blocks (Table 11). Reliance on clinical diagnosis in the absence of parasitological confirmation by microscopy or RDT is a major weakness to effective management of malaria in pregnancy in many study settings. Parasitological diagnosis was not always possible because of inadequate or lack of diagnostic facilities [15],[17],[21],[42],[43],[47] or prohibitive costs to women [17],[42]. One study noted concerns among providers about the poor predictive value of microscopy, with providers administering treatment to women with negative blood smears [52]. In Cambodia, monovalent RDTs that detected only *P. falciparum* were available at the village level; hence, people with a negative result were reported to self-treat for *P. vivax* [17].

**Table 11:** Content analysis of barriers to effective case management practices among healthcare providers.

Health Systems Building Blocks			Level							
			Individual		Organisational		Health System		Non-Health System	
			Quan	Qual	Quan	Qual	Quan	Qual	Quan	Qual
Governance/leadership	Documents	Lack of updated policy/training protocols					4	1		
	Supervision	Lack of supervision					3	4		
Health workforce/human resources	Cadre/persons	Insufficient number of staff					2	2		
	Training on case management	Insufficient training for diagnosis					0	2		
		Inadequate knowledge of treatment/confusion over guidelines					10	5		
Service delivery	Facility	Inadequate drug stocks			2	0				
	Diagnosis	Reliance on clinical symptoms	9	4						
		Inadequate facilities for diagnostic procedures					5	3		
	Treatment	Patient treatment preference							0	1
Health information systems	District level	Poor patient history records			1	1				
		Reliance on incomplete ANC cards			1	0				
Financing	Cost to user	Cost of diagnosis					1	1		
		Cost of treatment					5	1		
		Cost of maintained drug supplies			1	0				
	Cost to provider	Financial incentives to sell certain treatment brands	1	1						
Medical products and technology	Diagnosis	Low perceived efficacy of diagnostic techniques	1	1						
	Treatment	Low perceived efficacy of treatment options	5	1						
		Perception of safety of drugs during pregnancy	4	0						
		Fear of side effects in patient	6	2						
		Risk to patient due to age of patient							1	0
		Risk to patient due to gestational age							2	1
		Fear of growing antimalarial resistance	1	1						

Numbers indicate the number of studies included in this review that report each factor.

qual, qualitative; quan, quantitative.

Poor knowledge of and adherence to national treatment policy guidelines among healthcare providers was a consistent finding across countries in east [42],[51] and west Africa [21],[30],[37],[38],[40],[43],[44],[46],[47], Asia [16],[17], the Middle East [18], and Latin America [20], and knowledge was particularly poor among private providers [35],[39]. In Ghana, healthcare providers asserted that pregnant women were exceptions to the policy of testing prior to treatment, and provided treatment even when a malaria test was negative. This practice was a misinterpretation of the guidelines, which state that in the absence of a laboratory, pregnant women with clinical symptoms of malaria should be treated [21]. Financial incentives and client demand have been reported to motivate private practitioners to sell medicines, even inappropriate medicines or more expensive brands [40], without parasitological testing [17]. Prescription practices were influenced by perceptions of low drug efficacy [20],[37],[38],[46], perceptions of increasing drug resistance [17],[38], concerns about drug safety [35],[37],[46], inadequate understanding and fear of potential side effects of drugs in pregnancy [17],[35],[40],[46],[47],[51], and the influence of patient preference [17], each contributing to poor quality of care. Many of the substandard practices reported are a consequence of factors operating at higher levels of the health system. Lack of national guidelines in India led to healthcare provider confusion about treatment policy [15]. Elsewhere, lack of training for diagnosis [16],[20] and/or treatment [18],[20],[41]–[43],[51] and lack of supervision [16],[41],[47] contributed to poor service delivery. Poor provider knowledge was exacerbated by weak organisation at the level of the health facility, such as inadequate drug stocks [15], and poor record-keeping practices such as providing written prescriptions and infrequent recording of vital information [20] and clinical findings and diagnoses [36] in both in-patient and out-patient records. Health system policies on fees for diagnosis [17],[42] and prescription drugs [17],[30],[38],[41],[42],[46] and human resource constraints [41],[47] also constitute important barriers.

#### Determinants of knowledge and diagnostic and treatment practices among healthcare providers

Determinants of healthcare provider knowledge, diagnostic practices, and treatment practices explored across the different studies included the following: individual healthcare provider factors (cadre, training), type of facility (public or private; primary or tertiary), and region/location (Table 12). There was insufficient uniformity of indicators and determinants to perform a meta-analysis of pooled data. Cadre of healthcare provider was associated with correct treatment knowledge [47] and diagnostic practices [38]. Public sector providers were more likely to use clinical diagnosis or parasitological diagnosis with RDTs and to adhere to national guidelines than private providers in Nigeria [39]. Two studies assessed the impact of training on provider knowledge, finding a significant positive association between provider knowledge and recent training on malaria treatment guidelines [18],[47].

**Table 12:** Determinants affecting provider knowledge of malaria in pregnancy, diagnostic practices, and treatment practices.

Factor	Determinant	Study	Country	Scale	*N*	Effect Measure	Details
Healthcare provider knowledge	Cadre	Smith Paintain 2011 [47]	Ghana	7 districts	134	RR	Cadre of staff was not associated with level of knowledge of national treatment guidelines
							Those responsible for writing prescriptions were more likely to have correct knowledge of treatment policy for 2nd and 3rd trimesters than those of lower cadres (*p* = 0.06)
		Harrison 2012 [43]	Nigeria		123	Chi2	Cadre of doctor was not associated with awareness of malaria in pregnancy treatment guidelines
		Omo-Aghoja 2008 [37]	Nigeria	National	84	Chi2	Neither level of specialty training nor number of years in practice were associated with knowledge of national guidelines on treatment and prevention with IPTp (*p*>0.05)
		Kamuhabwa 2011 [35]	Tanzania	<1 district	200	Chi2	No difference in knowledge regarding contraindications of AL in pregnancy between pharmacist and non-pharmaceutical personnel
							No difference between pharmacists and non-pharmaceutical personnel concerning knowledge of: quinine, SP, DHA-PPQ; AQ; sulphalene-pyrimethamine
		Kiningu 2013 [41]	Kenya	<1 district	36	Fisher test	No difference in awareness or use of malaria in pregnancy clinical guidelines among professional cadres, education levels, or differences in duration of experience (*p*>0.05)
	Public or private	Onwujekwe 2012 [39]	Nigeria	<1 district	52	Chi2	No difference between public or private providers in reporting malaria in pregnancy as a serious condition (*p*>0.05)
	Training received	Smith Paintain 2011 [47]	Ghana	7 districts	134	RR	Recent attendance at training session resulted in greater knowledge of malaria in pregnancy treatment guidelines (1st trimester, *p* = 0.02; 2nd and 3rd trimesters, *p* = 0.04)
Diagnostic practices	Cadre	Umar 2011 [38]	Nigeria	1 state	25	Chi2	Exclusive use of clinical features to diagnose malaria in pregnancy was more frequently observed among staff with lower qualifications in primary health centres (*p* = 0.027)
	Public or private	Onwujekwe 2012 [39]	Nigeria	<1 district	52	Chi2	More public than private providers used symptom recognition to diagnose malaria in pregnancy (*p* = 0.02)
							No difference in use of microscopy to diagnose malaria in pregnancy between public and private providers
							More public than private providers used RDTs to diagnose malaria in pregnancy (*p* = 0.04)
	Training received	Bin Ghouth 2013 [18]	Yemen	3 districts	86	Chi2	HP training improved the frequency of prescription for quinine use in malaria in pregnancy from 17% to 60% (OR 4.9, *p* = 0.004) and reduced the use of artemether from 47% to 19% (OR 0.26, *p* = 0.01)
		Smith Paintain 2011 [47]	Ghana	7 districts	134	RR	Attendance at a malaria diagnosis workshop was not significantly associated with correct knowledge of treatment for policy for any trimester
	Regional differences	Wylie 2010 [15]	India	2 states	280	Chi2	Between regions, more providers in Chattisgargh used a combination of a presence of fever, blood smear microscopy, signs of anaemia, and haemoglobin levels to diagnose malaria in pregnancy (*p*<0.001)
Treatment practices	Type of health facility	Onwujekwe 2012 [39]	Nigeria	<1 district	52	Chi2	More public than private providers prescribed quinine in 1st trimester (*p* = 0.01)
							No difference in prescription of CQ between public and private providers
							More private than public providers prescribed SP for the treatment of malaria in pregnancy (*p*<0.001)
		Luz 2013 [19]	Brazil	>1 district	262	Chi2	No difference in treatment regimens or in prescriptions containing first choice antimalarials between reference centres for malaria and primary care units (*p*>0.05)

Chi2, Chi squared test; DHA-PPQ, dihydroartemisinin-piperaquine combination; HP, healthcare provider; OR, odds ratio; RR, adjusted risk ratio.

#### Intervention studies

Only one intervention study was identified, which evaluated the effect of in-service training of clinicians and pharmacists in the private sector in three governorates in Yemen on malaria treatment in pregnant women [18]. The post-training assessment showed improved knowledge of correct dosing, from 17% to 60%, still far short of 100%.

## Discussion

To our knowledge this review draws together for the first time findings from disparate studies on the treatment-seeking practices for malaria in pregnant women and the case management practices of a range of healthcare providers globally. The key emerging themes are relatively consistent across a range of study settings in terms of local cultural, socio-economic, health system, and non–health system contexts, and geographical locations. One- to three-quarters of women reported malaria illness during pregnancy, of whom treatment was sought by >85%. Self-medication and traditional healers were reportedly used by 5% to 40% of women, alongside care from the formal health sector (range 42%–92%). Knowledge of drug safety, cost, and perceptions of healthcare services affected treatment choices. Determinants of treatment seeking were education and prior experience of miscarriage or ANC use. Healthcare providers' reliance on clinical diagnosis and poor adherence to treatment guidelines by trimester were consistently reported. Prescribing practices were driven by poor knowledge of national guidelines and concerns over side effects and drug safety, patient preference, drug availability, and cost. Determinants of provider practices were individual provider factors (cadre, training), facility type (public or private; primary or tertiary), and sub-national region.

**Figure 5:**
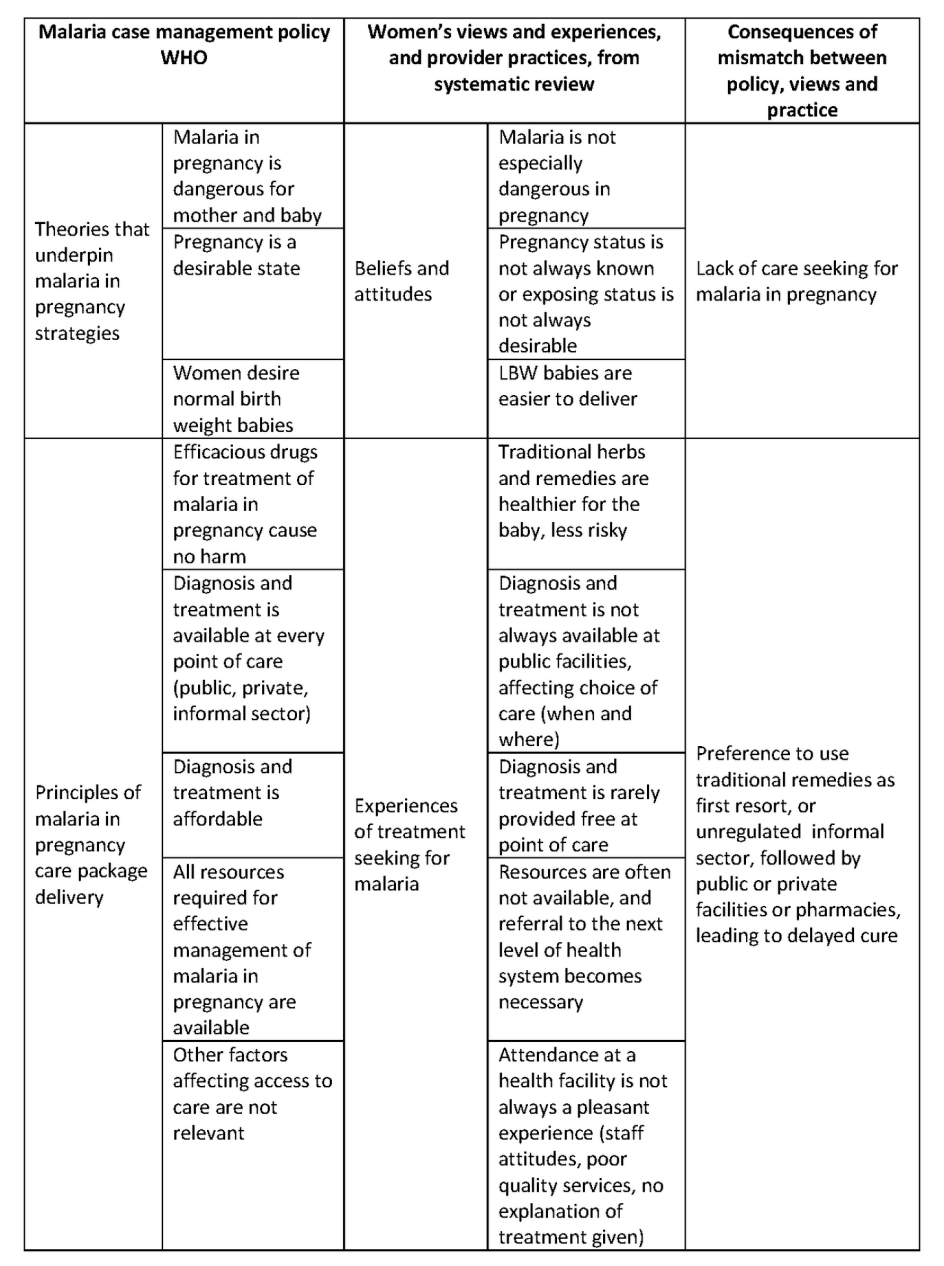
Integration of findings in relation to WHO case management policy [1]. LBW, low birth weight.

The review highlights important limitations in the implementation of the WHO policy on treatment of malaria in pregnancy [1],[2]. There is an apparent disconnect between the theories that underpin WHO policy and the beliefs and attitudes of women, in addition to which there is dissonance between the principles of delivery of quality care, and the experiences and practices of pregnant women and healthcare providers (Figure 5). Importantly, women do not uniformly seek care within the formal health system, and when they do, they may not access appropriate diagnosis and treatment, because of poor healthcare provider skills or inadequate resources or because they cannot afford to pay for the services.

Barriers to access among women—in particular, poor knowledge of drug safety, prohibitive costs, and self-treatment practices—suggest ministries of health need to improve women's access to information so that they can make informed choices. Educating women about the risks of malaria in pregnancy will be important, especially as the manifestation of malaria illness in pregnancy may be confused with pregnancy-related symptoms [53]. Even when women think they have malaria, a series of socio-cultural factors impede women's ability to act, such as not wishing to disclose their pregnancy status and other factors operating at the household level that restrict women's autonomy to seek care, as reported by others [17],[53],[54]. The finding that pregnant women may self-treat for malaria was partly attributed by the reviewed studies to the irregular and inadequate supply of drugs at health facilities. Given that alternative care providers include shop vendors (who are poorly informed or otherwise incentivised to give more expensive and sometimes inappropriate drugs) and TBAs (who practice and promote herbal remedies and traditional herbs as healthier or less risky to the developing foetus) [45], women need appropriate information about which antimalarials are recommended and safe to use for the treatment of malaria at different stages of pregnancy. Advertising provides an opportunity for governments to add messages about safety and use of antimalarials in pregnancy, and information pamphlets could be given to women at ANC facilities. One of the main barriers to women seeking treatment for malaria at formal health facilities is cost, both direct costs such as user fees and indirect costs, as has been reported in studies on antenatal care seeking more broadly [55],[56]. The economic barriers to accessing expensive diagnostic tests and treatment for malaria among pregnant women have been reported previously [57] and warrant a review of strategies to reach pregnant women with safe and affordable treatment options.

Diagnosis of malaria in pregnancy in many settings is challenging. In stable and high transmission settings, parasitological diagnosis based on microscopy or RDTs potentially reduces the unnecessary use of antimalarials in pregnancy, particularly in areas of high HIV prevalence, where HIV-infected patients have a high incidence of febrile illness [2]. As our review shows, the reality in resource-constrained public health facilities and among community providers is that diagnosis is frequently restricted to clinical symptoms. As observed in a clinical study among pregnant women in Mozambique, the positive predictive values of the three most common malaria symptoms—headache, arthromyalgias, and history of fever—for malaria parasitaemia were low (28%, 29%, and 33%, respectively) [58]. Even where diagnostic tests are available, studies reported that providers sometimes choose to ignore negative test results and prescribe antimalarials when malaria is suspected. The reasons for ignoring diagnostic test results are likely to be a combination of factors related to user preferences and demand, suspected insensitivity or inferiority of the tests, inferior reagents, or lack of trust in the accuracy of slide reading by laboratory staff. Efforts are needed to scale up the availability of RDTs at points of care used by pregnant women and to improve provider proficiency in their application.

Healthcare providers from a range of countries and continents and across a variety of cadres in the private and public sectors, and formal and informal sectors, demonstrated poor knowledge of and adherence to national treatment policy guidelines. Poor knowledge of and availability of treatment guidelines, and concerns over side effects and drug safety suggest the need for refresher training, job aids, and improved supervision. The finding that doctors were less likely to prescribe correctly may reflect personal judgements based on knowledge or individual client needs, as noted in a study in Zimbabwe where many practitioners felt that guidelines would limit their personal flexibility in caring for patients [59]. Health system strengthening is needed to improve drug availability, as well as legislation to promote rational drug use to eliminate the use of monotherapies and other non-recommended antimalarials across all service providers. Efforts by ministries of health to incorporate private sector providers into centralised training and dissemination activities on national treatment policy are needed. In addition, a licensing system to regulate which antimalarials are sold at the community level is needed to prohibit the use of monotherapies and to reduce women's exposure to ineffective drugs and the potential risks of ACT use in the first trimester.

With the advent of the 2006 WHO policy [2], inadvertent exposure to ACTs among pregnant women in the first trimester has been a considerable public health concern [3],[60]. Few studies in our review stratified antimalarial use by trimester; of those that did, prescription of ACTs in the first trimester was reported in Ghana, CAR, and Nigeria. This is a very real concern, and research by the Malaria in Pregnancy Consortium is ongoing to develop pharmacovigilance systems that can be implemented in resource-poor countries to monitor the safety of antimalarials in pregnancy, including inadvertent exposures in the early first trimester [61]. The continued use of drugs that are no longer recommended in national treatment policies, such as SP (recommended for IPTp only) in Ghana and Nigeria, and CQ in Nigeria (because of known high levels of parasite resistance to CQ) [62], is another area for concern. The use of artemisinin monotherapies is a major threat for the development of artemisinin resistance in the Africa region, as occurred in parts of Asia [63],[64].

The dearth of implementation research on interventions to improve the quality of case management of malaria in pregnancy underscores the fact that this is a neglected area of research, despite case management constituting one of the three key strategies for controlling malaria in pregnancy in sub-Saharan Africa, and a lifesaving intervention for both mother and child in lower transmission settings in Asia and Latin America. Research using standardised methodologies is needed to systematically document treatment seeking in pregnant women and healthcare provider practices across a range of countries and settings. Implementation research is needed to evaluate the impact of strengthened public sector practices on pregnant women's access to malaria treatment in the public sector, as well as strategies that target private drug sellers, such as better information, communication, and legislation for rational drug use.

### Strengths and Limitations

The review uses data from quantitative, qualitative, and mixed methods studies to increase the comprehensiveness of the review; studies with quantitative data provided frequencies of practices, the qualitative data provided important explanatory factors driving those behaviours, and the content analysis was useful to determine the frequency of reporting of the different factors associated with case management of malaria in pregnancy across studies. We did not attempt a meta-ethnography, as has been done by others [53]. The primary geographic scope of the review is Africa, since this is where the majority of the included studies were undertaken, with few available studies in Asia and Latin America. Whilst no restrictions were placed on the language, and no studies were excluded on the basis of language, the focus of the Malaria in Pregnancy Library (the primary source of studies) to date has been the European family of languages, predominantly English. Reviewer bias was limited by the use of two reviewers to independently assess inclusion criteria. The reporting of the included studies was assessed for quality, and reporting quality for the majority of studies was assessed to be moderate to high. Findings from five studies [12],[35],[44],[51],[52] assessed to be of low quality (meeting <50% of the quality criteria) were consistent with the other studies. Inconsistency in study methodologies and end points precluded a meta-analysis of pooled data of the determinants of women's access to treatment or healthcare provider case management practices. In the meta-analysis for source of treatment, there may have been overlap between sources of treatment reported, e.g., a doctor may practice in a health facility, antenatal clinic, or private clinic.

The majority of studies of women were undertaken at sub-district, district, or state level, which limits the generalisability of the individual studies. There was reasonable consistency of findings across different studies in the same country and across studies in different countries. The studies of healthcare providers had greater geographic scope, with eight of 18 studies undertaken in more than one district or state, three of which were done at national level.

## Conclusions

Our review highlights the poor quality of case management practices for malaria in pregnancy across many parts of Africa, Asia, and Latin America. These practices not only threaten the health outcomes for mothers and their infants, but endanger the prospective useful life of several therapeutic drugs, in particular the artemisinins, through the continued use of monotherapies. The challenge for ministries of health will be the deployment of legislative and quality improvement interventions to reach the broad range of healthcare providers that administer antimalarial drugs in the community, in the private and public sectors as well as in the formal and informal sectors. Further implementation research using standardised methodologies is needed to systematically assess the extent of substandard case management practices at the national scale, to review how policies are implemented and disseminated by countries, and to assess practitioner and patient adherence. Research to evaluate targeted or multifaceted interventions aimed to improve the delivery of and access to quality case management services for pregnant women should be a priority.

## Supporting Information

Table S1 Search terms and databases used in the review.
(DOCX)

Table S2 Checklist for quality of reporting: quantitative studies.
(DOCX)

Table S3 Checklist for quality of reporting: qualitative studies.
(DOCX)

Table S4 Checklist for quality of reporting: mixed methods studies.
(DOCX)

Table S5 Sub-group analysis for source of treatment among pregnant women.
(DOCX)

Table S6 Sub-group analysis for adherence to treatment policy among health care providers.
(DOCX)

Text S1 PRISMA statement.
(DOC)

## Abbreviations

ACT: artemisinin-based combination therapy
AL: artemether-lumefantrine
ANC: antenatal care
AQ: amodiaquine
AS: artesunate
CAR: Central African Republic
CHW: community health worker
CQ: chloroquine
IDP: internally displaced person
IPTp: intermittent preventive treatment in pregnancy
RDT: rapid diagnostic test
SP: sulphadoxine-pyrimethamine
TBA: traditional birth attendant

## References

[1] World Health Organization (2010) Guidelines for the treatment of malaria, second edition. Geneva: World Health Organization.

[2] World Health Organization (2006) Guidelines for the treatment of malaria. Geneva: World Health Organization.

[3] Ward SA, Sevene EJ, Hastings IM, Nosten F, McGready R (2007) Antimalarial drugs and pregnancy: safety, pharmacokinetics, and pharmacovigilance. Lancet Infect Dis 7: 136–144. 17251084 10.1016/S1473-3099(07)70025-7

[4] Roll Back Malaria (2014) Global malaria action plan for a malaria-free world: Part II: the global strategy. 2. Overcoming malaria. Available: http://www.rollbackmalaria.org/gmap/2-2.html#. Accessed 20 May 2014.

[5] Hill J, Hoyt J, van Eijk AM, D'Mello-Guyett L, Ter Kuile FO, et al (2013) Factors affecting the delivery, access, and use of interventions to prevent malaria in pregnancy in sub-Saharan Africa: a systematic review and meta-analysis. PLoS Med 10: e1001488. 23935459 10.1371/journal.pmed.1001488 PMC3720261

[6] van Eijk AM, Hill J, Povall S, Reynolds A, Wong H, et al (2013) The Malaria in Pregnancy Library: a bibliometric review. Malar J 11: 362. 23110589 10.1186/1475-2875-11-362 PMC3522037

[7] EBSCO Information Services (2014) Global Health Database [database]. Available: http://www.ebscohost.com/corporate-research/global-health. Accessed 15 April 2014.

[8] International Network for the Rational Use of Drugs (2014) INRUD Bibliography [database]. Available: http://www.inrud.org/Bibliographies/INRUD-Bibliography.cfm. Accessed 7 April 2014.

[9] Adam T, Hsu J, de Savigny D, Lavis JN, Rottingen JA, et al (2012) Evaluating health systems strengthening interventions in low-income and middle-income countries: are we asking the right questions? Health Policy Plan 27 (Suppl 4)iv9–iv19. 23014156 10.1093/heapol/czs086

[10] de Savigny D, Webster J, Agyepong IA, Mwita A, Bart-Plange C, et al (2012) Introducing vouchers for malaria prevention in Ghana and Tanzania: context and adoption of innovation in health systems. Health Policy Plan 27 (Suppl 4)iv32–iv43. 23014151 10.1093/heapol/czs087

[11] World Health Organization (2009) Systems thinking for health systems strengthening. Geneva: World Health Organization.

[12] Maiga AS, Diakite M, Diaware A, Sango HA, Coulibaly CO (2010) [Pharmacovigilance and impact of intermittent preventive treatment with sulfadoxine-pyrimethamine for pregnant women in Selingue in Mali.]. Mali Med 25: 41–48. 21441083

[13] Deeks JJ, Higgins JPT, Altman DG, editors (2009) Analysing data and undertaking meta-analyses. In: Higgins JPT, Green S, editors. Cochrane handbook for systematic reviews of interventions. Chichester (UK): The Cochrane Collaboration.

[14] Sabin LL, Rizal A, Brooks MI, Singh MP, Tuchman J, et al (2010) Attitudes, knowledge, and practices regarding malaria prevention and treatment among pregnant women in Eastern India. Am J Trop Med Hyg 82: 1010–1016. 20519593 10.4269/ajtmh.2010.09-0339 PMC2877404

[15] Wylie BJ, Hashmi AH, Singh N, Singh MP, Tuchman J, et al (2010) Availability and utilization of malaria prevention strategies in pregnancy in eastern India. BMC Public Health 10: 557. 20849590 10.1186/1471-2458-10-557 PMC2949771

[16] Population Services International Research and Metrics (2007) Cambodia 2007: TRaC study exploring the determinants of malaria health care provision among private providers in malaria endemic areas—first round. Washington (District of Columbia): Population Services International.

[17] Tawfik L (2006) Mosquitoes, malaria and malarine: a qualitative study on malaria drug use in Cambodia. Arlington (Virginia): US Agency for International Development.

[18] Bin Ghouth AS (2013) Availability and prescription practice of anti-malaria drugs in the private health sector in Yemen. J Infect Dev Ctries 7: 404–412. 23669430 10.3855/jidc.2528

[19] Luz TCB, Miranda ES, Freitas LF, Osorio-de-Castro CGS (2013) Prescriptions for uncomplicated malaria treatment among pregnant women in the Brazilian Amazon: evidences from the Mafalda Project. Rev Bras Epidemiol 16: 409–419. 24142012 10.1590/S1415-790X2013000200016

[20] Luz TC, Suarez-Mutis C, Miranda S, Moritz F, Freitas F, et al (2013) Uncomplicated malaria among pregnant women in the Brazilian Amazon: local barriers to prompt and effective case management. Acta Trop 125: 137–142. 23178220 10.1016/j.actatropica.2012.11.004

[21] Pell C, Meñaca A, Afrah NA, Manda-Taylor L, Chatio S, et al (2013) Prevention and management of malaria during pregnancy: findings from a comparative qualitative study in Ghana, Kenya and Malawi. Malar J 12: 427. 24257105 10.1186/1475-2875-12-427 PMC3874601

[22] Launiala A, Honkasalo ML (2010) Malaria, danger, and risk perceptions among the Yao in rural Malawi. Med Anthropol Q 24: 399–420. 20949843 10.1111/j.1548-1387.2010.01111.x

[23] Smith Paintain LA, Jones C, Adjei RO, Antwi GD, Afrah NA, et al (2010) Intermittent screening and treatment versus intermittent preventive treatment of malaria in pregnancy: user acceptability. Malar J 9: 18. 20074372 10.1186/1475-2875-9-18 PMC2817700

[24] Mbachu CO, Onwujekwe OE, Uzochukwu BS, Uchegbu E, Oranuba J, et al (2012) Examining equity in access to long-lasting insecticide nets and artemisinin-based combination therapy in Anambra state, Nigeria. BMC Public Health 12: 315. 22545723 10.1186/1471-2458-12-315 PMC3358243

[25] Sangaré LR, Weiss NS, Brentlinger PE, Richardson BA, Staedke SG, et al (2011) Patterns of anti-malarial drug treatment among pregnant women in Uganda. Malar J 10: 152. 21645402 10.1186/1475-2875-10-152 PMC3118160

[26] Henry OJ, Lagoro KD, Orach CG (2012) Prevalence of malaria and treatment seeking behaviours among pregnant women in postconflict internally displaced persons' camps in Gulu District. ISRN Public Health 2012: 164935 doi:10.5402/2012/164935

[27] Enato EF, Mens PF, Okhamafe AO, Okpere EE, Pogoson E, et al (2009) Plasmodium falciparum malaria in pregnancy: prevalence of peripheral parasitaemia, anaemia and malaria care-seeking behaviour among pregnant women attending two antenatal clinics in Edo State, Nigeria. J Obstet Gynaecol 29: 301–306. 19835496 10.1080/01443610902883320

[28] Obieche AO, Enato EF, Ande AB (2013) Patterns of treatment of reported malaria cases during pregnancy in a Nigerian hospital. Scand J Infect Dis 45: 849–854. 23968224 10.3109/00365548.2013.821205

[29] Onwujekwe O, Onwujekwe OO, Soremekun R (2013) Chemotherapy and chemoprophylaxis of malaria in pregnancy in private and public facilities: perceptions and use by pregnant women in Enugu State, Nigeria. Gend Behav 11: 5688–5697.

[30] Kwansa-Bentum B, Ayi I, Suzuki T, Otchere J, Kumagai T, et al (2011) Administrative practices of health professionals and use of artesunate-amodiaquine by community members for treating uncomplicated malaria in southern Ghana: implications for artemisinin-based combination therapy deployment. Trop Med Int Health 16: 1215–1224. 21740487 10.1111/j.1365-3156.2011.02833.x

[31] Adam I, Omer EsM, Salih A, Khamis A, Malik EM (2008) Perceptions of the causes of malaria and its complications, treatment and prevention among midwives and pregnant women of Eastern Sudan. J Public Health 16: 129–132.

[32] Karunamoorthi K, Deboch B, Tafere Y (2010) Knowledge and practice concerning malaria, insecticide-treated net (ITN) utilization and antimalarial treatment among pregnant women attending specialist antenatal clinics. J Public Health 18: 559–566.

[33] Sam-Wobo SO, Akinboroye T, Anosike JC, Adewale B (2008) Knowledge and practices on malaria treatment measures among pregnant women in Abeokuta, Nigeria. Tanzan J Health Res 10: 226–231. 19402584 10.4314/thrb.v10i4.45078

[34] Mbonye AK, Birungi J, Yanow S, Magnussen P (2013) Prescription patterns and drug use among pregnant women with febrile Illnesses in Uganda: a survey in out-patient clinics. BMC Infect Dis 13: 237. 23702003 10.1186/1471-2334-13-237 PMC3668983

[35] Kamuhabwa AR, Mnyusiwalla F (2011) Rational dispensing and use of artemether-lumefantrine during pregnancy in Dar es Salaam, Tanzania. Tanzan J Health Res 13. 10.4314/thrb.v13i2.63122 25566607

[36] Manirakiza A, Soula G, Laganier R, Klement E, Djalle D, et al (2011) Pattern of the antimalarials prescription during pregnancy in Bangui, Central African Republic. Malar Res Treat 2011: 414510. 22312567 10.4061/2011/414510 PMC3265284

[37] Omo-Aghoja LO, Aghoja CO, Oghagbon K, Omo-Aghoja VW, Esume C (2008) Prevention and treatment of malaria in pregnancy in Nigeria: obstetrician's knowledge of guidelines and policy changes-a call for action. J Chin Clin Med 3: 114–120.

[38] Umar MT, Chika A, Jimoh AO (2011) Compliance of primary health care providers to recommendation of artemesinin-based combination therapy in the treatment of uncomplicated malaria in selected primary health care centres in Sokoto, north western Nigeria. Int J Trop Med 6: 70–72.

[39] Onwujekwe OC, Soremekun RO, Uzochukwu B, Shu E, Onwujekwe O (2012) Patterns of case management and chemoprevention for malaria-in-pregnancy by public and private sector health providers in Enugu state, Nigeria. BMC Res Notes 5: 211. 22551039 10.1186/1756-0500-5-211 PMC3392746

[40] Okoro RN, Nwambu JO (2012) Evaluation of physicians' prescribing patterns of antimalarial drugs during pregnancy at the obstetrics and gynaecology department of a teaching hospital in Maduguri, Borno State, Nigeria. Int J Pharm Biomed Sci 3: 39–46.

[41] Kiningu DK (2013) Factors influencing the use of evidence based guidelines in the management of malaria in pregnancy among health workers at Garissa Provincial Hospital, Kenya [Master's thesis]. Nairobi: School of Public Health, University of Nairobi. Available: http://erepository.uonbi.ac.ke:8080/xmlui/bitstream/handle/11295/59598/Factors%20Influencing%20The%20Use%20Of%20Evidence%20Based%20Guidelines%20in%20the%20Management%20of%20Malaria%20in%20Pregnancy%20Among%20Health%20Workers%20at%20Garissa%20Provincial%20Hospital%20Kenya.pdf?sequence=3. Accessed 7 July 2014

[42] Kalilani-Phiri LV, Lungu D, Coghlan R (2011) Knowledge and malaria treatment practices using artemisinin combination therapy (ACT) in Malawi: survey of health professionals. Malar J 10: 279. 21939507 10.1186/1475-2875-10-279 PMC3196928

[43] Harrison N, Olufunlayo T, Agomo C (2012) Utilization of the current national antimalarial treatment guidelines among doctors in army hospitals in Lagos, Nigeria. Open J Prev Med 2: 390–393.

[44] Enato EFO, Erihri RE (2012) Knowledge, perception and management of malaria in pregnancy by traditional birth attendants in Benin City. J Pharm Allied Sci 8: 1292–1297.

[45] Stangeland T, Alele PE, Katuura E, Lye KA (2011) Plants used to treat malaria in Nyakayojo sub-county, western Uganda. J Ethnopharmacol 137: 154–166. 21575702 10.1016/j.jep.2011.05.002

[46] Okonta PI (2011) How many physicians prescribe quinine for the treatment of malaria in the first trimester of pregnancy? Ebonyi Med J 10: 105–111.

[47] Smith Paintain L, Antwi GD, Jones C, Amoako E, Adjei RO, et al (2011) Intermittent screening and treatment versus intermittent preventive treatment of malaria in pregnancy: provider knowledge and acceptability. PLoS ONE 6: e24035. 21887367 10.1371/journal.pone.0024035 PMC3161113

[48] Malawi Ministry of Health (2008) Republic of Malawi Ministry of Health national malaria control program supervision report for monitoring ACT and malaria control activities. Lilongwe: Malawi Ministry of Health.

[49] Uganda Ministry of Health (2005) National policy on malaria treatment 2005. Kampala: Uganda Ministry of Health.

[50] Tanzania Ministry of Health and Social Welfare (2006) National guidelines for malaria diagnosis and treatment 2005. Dar es Salaam: Tanzania Ministry of Health and Social Welfare.

[51] Minyaliwa C, Bandawe C, Mwale RJ (2012) How much do Blantyre dispensers in hospital and community pharmacies know about the new malaria treatment guidelines? Malawi Med J 24: 1–4. 23638259 PMC3588198

[52] Mbonye AK, Magnussen P (2010) Symptom-based diagnosis of malaria and its implication on antimalarial drug use in pregnancy in central Uganda: results from a community trial. Int J Adolesc Med Health 22: 257–262. 21061926 10.1515/ijamh.2010.22.2.257

[53] Pell C, Straus L, Andrew EVW, Meñaca A, Pool R (2011) Social and cultural factors affecting uptake of interventions for malaria in pregnancy in Africa: a systematic review of the qualitative research. PLoS ONE 6: e22452. 21799859 10.1371/journal.pone.0022452 PMC3140529

[54] Finlayson K, Downe S (2013) Why do women not use antenatal services in low- and middle-income countries? A meta-synthesis of qualitative studies. PLoS Med 10: e1001373. 23349622 10.1371/journal.pmed.1001373 PMC3551970

[55] Johnson A, Goss A, Beckerman J, Castro A (2012) Hidden costs: the direct and indirect impact of user fees on access to malaria treatment and primary care in Mali. Soc Sci Med 75: 1786–1792. 22883255 10.1016/j.socscimed.2012.07.015

[56] Perkins M, Brazier E, Themmen E, Bassane B, Diallo D, et al (2009) Out-of-pocket costs for facility-based maternity care in three African countries. Health Policy Plan 24: 289–300. 19346273 10.1093/heapol/czp013 PMC2699243

[57] Worrall E, Morel C, Yeung S, Borghi J, Webster J, et al (2007) The economics of malaria in pregnancy—a review of the evidence and research priorities. Lancet Infect Dis 7: 156–168. 17251086 10.1016/S1473-3099(07)70027-0

[58] Bardaji A, Sigauque B, Bruni L, Romagosa C, Sanz S, et al (2008) Clinical malaria in African pregnant women. Malar J 7: 27. 18234078 10.1186/1475-2875-7-27 PMC2267805

[59] Bhagat K, Nyazema N (2001) General practitioners and clinical guidelines. East Afr Med J 78: 30–34. 11320762 10.4314/eamj.v78i1.9109

[60] Crawley J, Hill J, Yartey J, Robalo M, Serufilira A, et al (2007) From evidence to action? Challenges to policy change and programme delivery for malaria in pregnancy. Lancet Infect Dis 7: 145–155. 17251085 10.1016/S1473-3099(07)70026-9

[61] Dellicour S, ter Kuile FO, Stergachis A (2008) Pregnancy exposure registries for assessing antimalarial drug safety in pregnancy in malaria-endemic countries. PLoS Med 5: e187. 18788893 10.1371/journal.pmed.0050187 PMC2531138

[62] Bloland P (2001) Drug resistance in malaria. WHO/CDS/CSR/DRS/2001.4. Geneva: World Health Organization.

[63] World Health Organization (2005.) Global report on antimalarial drug efficacy and drug resistance: 2000–2010. Geneva: World Health Organization.

[64] Fairhurst RM, Nayyar GM, Breman JG, Hallett R, Vennerstrom JL, et al (2012) Artemisinin-resistant malaria: research challenges, opportunities, and public health implications. Am J Trop Med Hyg 87: 231–241. 22855752 10.4269/ajtmh.2012.12-0025 PMC3414557

