# Supplementary material for: Women's Access and Provider Practices for the Case Management of Malaria during Pregnancy: A Systematic Review and Meta-Analysis
Source: PLoS Med. 2014 Aug 5;11(8):e1001688. doi: 10.1371/journal.pmed.1001688 (PMC4122360; doi:10.1371/journal.pmed.1001688)
Supplement: Table S1 — Search terms and databases used in the review. (DOCX) [file pmed.1001688.s001.docx]

**Table S1. Search terms and databases used in the review.**

| **Search terms for Pregnant Women** | |  |
| --- | --- | --- |
| **Global Health Search** | **MiP Library Search*** | **INRUD*** |
| pregnant wom*  **AND** | ---- | pregnant wom*  **AND** |
| malaria  **AND** | ---- | malaria |
| treat* OR treatment seeking behav* OR health seeking behav* OR care seeking behav* OR treatment seeking practice* OR health seeking practice* OR care seeking practice* OR treatment seeking decision*  **AND** | treat* OR treatment seeking behav* OR health seeking behav* OR care seeking behav* OR treatment seeking practice* OR health seeking practice* OR care seeking practice* OR treatment seeking decision*  **AND** | ---- |
| determinant* OR factor* OR knowledge OR attitude* OR practice* OR compliance OR adherence OR frequenc* OR symptom* OR recogn* OR perception | determinant* OR factor* OR knowledge OR attitude* OR practice* OR compliance OR adherence OR frequenc* OR symptom* OR recogn* OR perception | ---- |

| **Search terms for Health Provider** | |  |
| --- | --- | --- |
| **Global Health Search** | **MiP Library Search*** | **INRUD** |
| pregnan* OR pregnant wom*  **AND** | ---- | pregnant wom*  **AND** |
| provider* OR ANC service* OR antenatal service* OR ANC OR health provider OR health work* OR health servic* provider OR drug shop vendor OR community health worker OR licensed chemical seller OR patent medicine seller OR ADDO OR community drug dispenser OR formal service OR informal service  **AND** | provider* OR ANC service* OR antenatal service* OR ANC OR health provider OR health work* OR health servic* provider OR drug shop vendor OR community health worker  **AND** | malaria |
| malaria  **AND** | ---- | ---- |
| case management OR control OR management OR treat* OR diagno* OR prescrib* OR practice* OR chloroquine OR CQ OR quinine OR ACT OR artemisinin-based combination therapy OR safe* OR community case management OR refer* OR practice  **AND** | case management OR control OR management OR treat* OR diagno* OR prescrib* OR practice* OR safe* OR community case management OR refer* OR practice  **AND** | ---- |
| knowledge OR compliance OR adherence OR proportion* OR frequenc* OR symptom* OR refer* | knowledge OR compliance OR adherence OR proportion* OR frequenc* OR symptom* OR refer* |  |

1* All material in the MiP Library contain the words *malaria* and *pregnant women*

2* Terms kept very broad for INRUD search
