# Supplementary material for: Women's Access and Provider Practices for the Case Management of Malaria during Pregnancy: A Systematic Review and Meta-Analysis
Source: PLoS Med. 2014 Aug 5;11(8):e1001688. doi: 10.1371/journal.pmed.1001688 (PMC4122360; doi:10.1371/journal.pmed.1001688)
Supplement: Table S2 — Checklist for quality of reporting: quantitative studies. (DOCX) [file pmed.1001688.s002.docx]

**Table S2. Checklist for quality of reporting: Quantitative Studies.**

We appraised the quality of reporting of each study using a checklist of criteria based on methods described in a previous review [[1](#_ENREF_1)]. Quantitative studies were assessed for reporting of 10 criteria, as follows: study context, sampling strategy, use of randomization, methodology, systematic data analysis, multivariate analysis, the minimization of recall, social desirability and measurement bias, and whether the findings were discussed in reference to policy, programming or further research [[2](#_ENREF_2),[3](#_ENREF_3)].

|  | **Criteria** | | | | | | | | | | **SCORE (n/10)** |
| --- | --- | --- | --- | --- | --- | --- | --- | --- | --- | --- | --- |
| **Author/Year** | **Description of Context** | **Participants and Sampling described** | **Randomiza-tion used** | **Methods described** | **Systematic Data Analysis described** | **Multivariate analysis used** | **Recall bias minimised** | **Social desirability bias minimised** | **Measurement bias minimised** | **Findings discussed** |  |
| Adam, 2008 [[4](#_ENREF_4)] | √ | √ |  | √ | √ |  |  |  |  | √ | 5 |
| Enato, 2009 [[5](#_ENREF_5)] | √ | √ | √ | √ | √ | √ |  |  | √ | √ | 7 |
| Ghouth, 2013 [[6](#_ENREF_6)] | √ | √ |  | √ | √ |  | √ |  |  | √ | 6 |
| Harrison, 2012 [[7](#_ENREF_7)] | √ | √ |  | √ | √ |  |  |  |  | √ | 5 |
| Henry. 2012 [[8](#_ENREF_8)] | √ | √ | √ | √ | √ |  | √ | √ | √ | √ | 9 |
| Kalilani-Phiri, 2011 [[9](#_ENREF_9)] | √ | √ | √ | √ | √ |  |  | √ | √ | √ | 8 |
| Kamuhabwa, 2011[[10](#_ENREF_10)] | √ | √ |  | √ | √ |  |  |  |  |  | 4 |
| Karunamoorthi, 2010 [[11](#_ENREF_11)] | √ | √ |  | √ | √ |  |  |  | √ | √ | 6 |
| Kwansa-Bentum, 2011 [[12](#_ENREF_12)] | √ | √ | √ | √ | √ |  |  | √ | √ | √ | 8 |
| Luz, 2013 A [[13](#_ENREF_13)] | √ | √ |  | √ | √ |  | √ | √ |  | √ | 7 |
| Maiga, 2010 [[14](#_ENREF_14)] |  | √ |  | √ | √ |  |  |  |  | √ | 4 |
| Manirakiza, 2011 [[15](#_ENREF_15)] | √ | √ |  | √ | √ |  | √ | √ | √ | √ | 8 |
| Mbachu, 2012 [[16](#_ENREF_16)] | √ | √ | √ | √ | √ | √ | √ |  | √ | √ | 9 |
| Mbonye, 2010 [[17](#_ENREF_17)] |  | √ | √ | √ | √ |  |  |  |  |  | 4 |
| Mbonye, 2013 [[18](#_ENREF_18)] | √ | √ |  | √ | √ |  |  |  | √ | √ | 6 |
| Minyaliwa, 2012 [[19](#_ENREF_19)] |  | √ |  | √ | √ |  |  |  |  | √ | 4 |
| Obieche, 2013 [[20](#_ENREF_20)] | √ | √ |  | √ | √ |  | √ | √ |  | √ | 7 |
| Okonta, 2011 [[21](#_ENREF_21)] | √ | √ |  | √ | √ |  | √ | √ | √ | √ | 8 |
| Okoro, 2012 [[22](#_ENREF_22)] | √ | √ | √ | √ | √ |  |  |  |  | √ | 6 |
| Omo-Aghoja, 2008 [[23](#_ENREF_23)] | √ | √ |  | √ | √ |  | √ | √ | √ | √ | 8 |
| Onwujekwe, 2012 [[24](#_ENREF_24)] | √ | √ |  | √ | √ |  | √ | √ | √ | √ | 8 |
| Onwujekwe, 2013 [[25](#_ENREF_25)] | √ | √ |  | √ | √ |  |  |  |  | √ | 5 |
| PSI Cambodia, 2007 [[26](#_ENREF_26)] | √ | √ | √ | √ | √ |  |  |  |  | √ | 6 |
| Sam-Wobo, 2008 [[27](#_ENREF_27)] | √ | √ |  | √ | √ |  |  |  |  | √ | 5 |
| Sangare, 2011 [[28](#_ENREF_28)] | √ | √ | √ | √ | √ |  | √ | √ | √ | √ | 9 |
| Umar, 2011 [[29](#_ENREF_29)] |  | √ | √ | √ | √ |  |  |  |  | √ | 5 |
| Wylie, 2010 [[30](#_ENREF_30)] | √ | √ |  | √ | √ |  |  |  |  | √ | 5 |

| **Description of categories:** √ **indicates it was reported in the article** | |
| --- | --- |
| **Description of context** | Authors report an adequate description of setting (urban/rural), time of study and location |
| **Participants and sampling described** | Authors report sampling methods, details of participants and randomization is discussed |
| **Randomization used** | Authors report use of randomization in sampling technique |
| **Methods described** | Authors use appropriate methods to address aims of study, provide detailed research procedures, express expertise amongst the research team to conduct methods, or report training of facilitators |
| **Systematic data analysis described** | Authors provide a detailed procedure of analysis, with justification for the method of analysis |
| **Multivariate analysis used** | Authors report use of multivariate analysis to control for confounding |
| **Recall bias minimised** | Authors report using methods to reduce recall bias (e.g. use of hospital records etc. rather than memory) |
| **Social desirability bias minimised** | Authors report use of methods to reduce social desirability (e.g. stock checks, check HCF records, check storage of medicines, check for ITN) |
| **Measurement bias minimised** | Authors report on the role of researcher, the relationship of researcher to participants/context, adequate training of staff, use of standardized research tools, and the use of standardized measurements |
| **Findings discussed** | Authors report the findings/results in terms of their impact on further research, programming and policy |

**References**

1. Hill J, Hoyt J, van Eijk AM, D'Mello-Guyett L, Ter Kuile FO, et al. (2013) Factors affecting the delivery, access, and use of interventions to prevent malaria in pregnancy in sub-Saharan Africa: a systematic review and meta-analysis. PLoS Med 10: e1001488.

2. Sanderson S, Tatt LD, Higgins JPT (2007) Tools for assessing quality and susceptibility to bias in observational studies in epidemiology: a systematic review and annotated bibliography. International Journal of Epidemiology 36: 666-676.

3. von Elm E, Altman DG, Egger M, Pocock SJ, Gotzsche PC, et al. (2007) The strengthening the reporting of observational studies in epidemiology (STROBE) statement: guidelines for reporting observational studies. Bulletin of the World Health Organization 85: 867-872.

4. Adam I, Omer el sir M, Salih A, Khamis A, Malik EM (2008) Perceptions of the causes of malaria and its complications, treatment and prevention among midwives and pregnant women of Eastern Sudan. Journal of Public Health: 129-132.

5. Enato E. F., Mens P. F., Okhamafe A. O., Okpere E. E., Pogoson E., et al. (2009) Plasmodium falciparum malaria in pregnancy: prevalence of peripheral parasitaemia, anaemia and malaria care-seeking behaviour among pregnant women attending two antenatal clinics in Edo State, Nigeria. Journal of Obstetrics and Gynaecology 29: 301-306.

6. Bin Ghouth A.S. (2013) Availability and prescription practice of anti-malaria drugs in the private health sector in Yemen. Journal of Infection in Developing Countries 7: 404-412.

7. Harrison N, Olufunlayo T, Agomo C (2012) Utilization of the current national antimalarial treatment guidleines among doctors in army hospitals in Lagos, Nigeria. Open Journal of Preventive Medicine 2: 390-393.

8. Henry O J., Lagoro K D., Orach C G. (2012) Prevalence of malaria and treatment seeking behaviours among pregnant women in postconflict internally displaced persons' camps in Gulu District. ISRN Public Health 2012: 164935. doi:10.5402/2012/164935.

9. Kalilani-Phiri LV., Lungu D., Coghlan R. (2011) Knowledge and malaria treatment practices using artemisinin combination therapy (ACT) in Malawi: survey of health professionals. Malaria Journal 10: 279.

10. Kamuhabwa AR, Mnyusiwalla F (2011) Rational dispensing and use of artemether-lumefantrine during pregnancy in Dar es Salaam, Tanzania. Tanzania Journal of Health Research 13.

11. Karunamoorthi K, Deboch B, Tafere Y (2010) Knowledge and practice concerning malaria, insecticide-treated net (ITN) utilization and antimalarial treatment among pregnant women attending specialist antenatal clinics. Journal of Public Health 18: 559-566.

12. Kwansa-Bentum B, Ayi I, Suzuki T, Otchere J, Kumagai T, et al. (2011) Administrative practices of health professionals and use of artesunate-amodiaquine by community members for treating uncomplicated malaria in southern Ghana: implications for artemisinin-based combination therapy deployment. Tropical Medicine and International Health 16: 1215-1224.

13. Luz TCB, Miranda ES, Freitas LF, Osorio-de-Castro CGS (2013 A) Prescriptions for uncomplicated malaria treatment among pregnant women in the Brazilian Amazon: evidences from the Mafalda Project. Revista Brasileira de Epidemiologia, 16: 409 - 419 PMID PM: 24142012.

14. Maiga AS, Diakite M, Diaware A, Sango HA, Coulibaly CO (2010) Pharmacovigilance and impact of intermittent preventive treatment with sulfadoxine-pyrimethamine for pregnant women in Selingue in Mali. Mali Medical 25: 41-48.

15. Manirakiza A, Soula G, Laganier R, Klement E, Djalle D, et al. (2011) Pattern of the antimalarials prescription during pregnancy in Bangui, Central African Republic. Malaria Research and Treatment 2011: 414510.

16. Mbachu CO, Onwujekwe O. E., Uzochukwu B. S., Uchegbu E., Oranuba J., et al. (2012) Examining equity in access to long-lasting insecticide nets and artemisinin-based combination therapy in Anambra state, Nigeria. BMC Public Health 12: 315.

17. Mbonye AK, Magnussen P (2010) Symptom-based diagnosis of malaria and its implication on antimalarial drug use in pregnancy in central Uganda: results from a community trial. International Journal of Adolescent Medicine and Health 22: 257-262.

18. Mbonye AK, Birungi J, Yanow S, Magnussen P (2013) Prescription patterns and drug use among pregnant women with febrile Illnesses in Uganda: a survey in out-patient clinics. BMC Infectious Diseases 13: 237 PMID 23702003.

19. Minyaliwa C, Bandawe C, Mwale RJ (2012) How much do Blantyre dispensers in hospital and community pharmacies know about the new malaria treatment guidelines? Malawi Medical Journal 24: 1-4.

20. Obieche AO, Enato EF, Ande AB (2013) Patterns of treatment of reported malaria cases during pregnancy in a Nigerian hospital. Scandinavian Journal of Infectious Diseases 45:849-854. PMID 23968224.

21. Okonta PI (2011) How many physicians prescribe quinine for the treatment of malaria in the first trimester of pregnancy? . Ebonyi Medical Journal 10: 105-111.

22. Okoro RN, Nwambu JO (2012) Evaluation of physicians' prescribing patterns of antimalarial drugs during pregnancy at the obstetrics and gynaecology department of a teaching hospital in Maduguri, Borno State, Nigeria. International Journal of Pharmacy and BioMedical Sciences 3: 39-46.

23. Omo-Aghoja LO, Aghoja CO, Oghagbon K, Omo-Aghoja VW, Esume C (2008) Prevention and treatment of malaria in pregnancy in Nigeria: obstetrician's knowledge of guidelines and policy changes-a call for action. Journal of Chinese Clinical Medicine 3: 114-120.

24. Onwujekwe OC, Soremekun RO, Uzochukwu B, Shu E, Onwujekwe O (2012) Patterns of case management and chemoprevention for malaria-in-pregnancy by public and private sector health providers in Enugu state, Nigeria. BMC Research Notes 5: 211.

25. Onwujekwe O, Onwujekwe OO, Soremekun R (2013) Chemotherapy and chemoprophylaxis of malaria in pregnancy in private and public facilities: perceptions and use by pregnant women in Enugu State, Nigeria. Gender and Behaviour 11: 5688 - 5697.

26. PSI Cambodia (2007) Cambodia 2007: TRaC Study exploring the determinants of malaria health care provision among private providers in malaria endemic areas. First Round. Washington, District of Columbia

27. Sam-Wobo SO, Akinboroye T, Anosike JC, Adewale B (2008) Knowledge and practices on malaria treatment measures among pregnant women in Abeokuta, Nigeria. Tanzania Journal of Health Research 10: 226-231.

28. Sangare LR, Weiss NS, Brentlinger PE, Richardson BA, Staedke SG, et al. (2011) Patterns of anti-malarial drug treatment among pregnant women in Uganda. Malaria Journal 10: 152.

29. Umar MT, Chika A, Jimoh AO (2011) Compliance of primary health care providers to recommendation of artemesinin-based combination therapy in the treatment of uncomplicated malaria in selected primary health care centres in Sokoto, north western Nigeria. International Journal of Tropical Medicine 6: 70-72.

30. Wylie BJ, Hashmi AH, Singh N, Singh MP, Tuchman J, et al. (2010) Availability and utilization of malaria prevention strategies in pregnancy in eastern India. BMC Public Health 10: 557.
