# Supplementary material for: Women's Access and Provider Practices for the Case Management of Malaria during Pregnancy: A Systematic Review and Meta-Analysis
Source: PLoS Med. 2014 Aug 5;11(8):e1001688. doi: 10.1371/journal.pmed.1001688 (PMC4122360; doi:10.1371/journal.pmed.1001688)
Supplement: Table S3 — Checklist for quality of reporting: qualitative studies. (DOCX) [file pmed.1001688.s003.docx]

**Table S3. Checklist for quality of reporting: Qualitative Studies.**

We appraised the quality of reporting of each study using a checklist of criteria based on methods described in a previous review [[1](#_ENREF_1)]. Qualitative studies were assessed for the extent to which the authors described 8 criteria: study context, sampling strategy, methodology, saturation of data, systematic data analysis, the reflexivity of the researcher, the reliability and validity of findings, and the relevance of findings to policy, programming or further research [[2](#_ENREF_2),[3](#_ENREF_3)].

|  | **Criteria** | | | | | | | | **SCORE (n/8)** |
| --- | --- | --- | --- | --- | --- | --- | --- | --- | --- |
| **Author/Year** | **Description of Context** | **Participants and Sampling** | **Methods described** | **Saturation mentioned** | **Systematic Data Analysis described** | **Reliability and validity discussed** | **Reflexivity of researcher documented** | **Findings discussed** |  |
| Enato, 2012 [[4](#_ENREF_4)] |  | √ | √ |  | √ |  |  | √ | 4 |
| Lanuiala, 2010 [[5](#_ENREF_5)] | √ | √ | √ |  |  | √ |  | √ | 5 |
| Pell, 2013 [[6](#_ENREF_6)] | √ | √ | √ | √ | √ | √ | √ | √ | 8 |
| Smith-Paintain, 2010 [[7](#_ENREF_7)] | √ | √ | √ |  | √ | √ | √ | √ | 7 |

| **Description of categories:** √ **indicates it was reported in the article** | |
| --- | --- |
| **Description of context** | Authors report an adequate description of setting (urban/rural), time of study and location |
| **Participants and sampling described** | Authors report sampling methods, details of participants and randomization is discussed |
| **Methods described** | Authors use appropriate methods to address aims of study, provide detailed research procedures, express expertise amongst the research team to conduct methods, or report training of facilitators |
| **Saturation mentioned** | Authors discuss the saturation of data from using research methods chosen |
| **Systematic data analysis described** | Authors provide a detailed procedure of analysis, with justification for the method of analysis |
| **Reliability and validity discussed** | Authors can express reliability and report steps taken to reduce measurement, social desirability and measurement biases (e.g. triangulation of methods), and can describe validation of transcripts/data |
| **Reflexivity of researcher documented** | Authors report on the effect of the researcher, role of researcher and the relationship of researcher to participants/context |
| **Findings discussed** | Authors report the findings/results in terms of their impact on further research, programming and policy |

**References**

1. Hill J, Hoyt J, van Eijk AM, D'Mello-Guyett L, Ter Kuile FO, et al. (2013) Factors affecting the delivery, access, and use of interventions to prevent malaria in pregnancy in sub-Saharan Africa: a systematic review and meta-analysis. PLoS Med 10: e1001488.

2. Barbour RS (2001) Checklists for improving rigour in qualitative research: a case of the tail wagging the dog? BMJ 322: 1115-1117.

3. Mays N, Pope C (2000) Qualitative research in health care. Assessing quality in qualitative research. BMJ 320: 50-52.

4. Enato EFO, Erihri RE (2012) Knowledge, perception and management of malaria in pregnancy by traditional birth attendants in Benin City. Journal of Pharmaceutical and Allied Sciences 8: 1292-1297.

5. Launiala A, Honkasalo ML (2010) Malaria, danger, and risk perceptions among the Yao in rural Malawi. Medical Anthropology Quarterly Q24: 399-420.

6. Pell C, Menaca A, Afrah NA, Manda-Taylor L, Chatio S, et al. (2013) Prevention and management of malaria during pregnancy: findings from a comparative qualitative study in Ghana, Kenya and Malawi. Malaria Journal 12: 427. PMID PM:24257105.

7. Smith Paintain LA, Jones C, Adjei RO, Antwi GD, Afrah NA, et al. (2010) Intermittent screening and treatment versus intermittent preventive treatment of malaria in pregnancy: user acceptability. Malaria Journal 9: 18.
