# Supplementary material for: Women's Access and Provider Practices for the Case Management of Malaria during Pregnancy: A Systematic Review and Meta-Analysis
Source: PLoS Med. 2014 Aug 5;11(8):e1001688. doi: 10.1371/journal.pmed.1001688 (PMC4122360; doi:10.1371/journal.pmed.1001688)
Supplement: Table S4 — Checklist for quality of reporting: mixed methods studies. (DOCX) [file pmed.1001688.s004.docx]

**Table S4. Checklist for quality of reporting: Mixed Methods.**

We appraised the quality of reporting of each study using a checklist of criteria based on methods described in a previous review [[1](#_ENREF_1)]. Mixed methods studies were assessed for the reporting of 11 criteria: study context, sampling strategy, methodology, justification of mixed methods, systematic data analysis, multivariate analysis, minimization of bias, the integration of qualitative and quantitative findings and the extent to which the findings were discussed in reference to policy, programming or further research [[2-4](#_ENREF_2)].

|  | **Criteria** | | | | | | | | | | | **SCORE (n/11)** |
| --- | --- | --- | --- | --- | --- | --- | --- | --- | --- | --- | --- | --- |
| **Author/Year** | **Description of Context** | **Participants and Sampling described** | | **Methods described** | **Justification of mixed methods** | **Systematic Data Analysis described** | | **Multivariate analysis used** | **Minimization of bias discussed** | **Integration of QUAN/QUAL components** | **Findings discussed** |  |
|  |  | **QUAN** | **QUAL** |  |  | **QUAN** | **QUAL** |  |  |  |  |  |
| Kiningu, 2013 [[5](#_ENREF_5)] | √ | √ | √ | √ | √ | √ | √ |  | √ | √ | √ | 10 |
| Luz, 2013 B [[6](#_ENREF_6)] | √ | √ | √ | √ |  | √ | √ |  |  | √ | √ | 9 |
| Sabin, 2010 [[7](#_ENREF_7)] | √ | √ | √ | √ | √ | √ | √ |  | √ | √ | √ | 10 |
| Smith-Paintain, 2011 [[8](#_ENREF_8)] | √ | √ | √ | √ |  | √ | √ | √ | √ | √ | √ | 10 |
| Stangeland, 2011 [[9](#_ENREF_9)] | √ | √ | √ | √ | √ | √ | √ |  |  | √ | √ | 9 |
| Tawfik, 2006 [[10](#_ENREF_10)] | √ | √ | √ | √ |  | √ | √ |  | √ | √ | √ | 9 |

| **Description of categories:** √ **indicates it was reported in the article** | |
| --- | --- |
| **Description of context** | Authors report an adequate description of setting (urban/rural), time of study and location |
| **Participants and sampling described** | Authors report sampling methods, justify use of sampling methods and provide details of participants |
| **Methods described** | Authors use appropriate methods to address aims of study, provide detailed research procedures, express expertise amongst the research team to conduct methods, or report training of facilitators |
| **Justification of mixed methods** | Authors discuss and justify the purpose, priority and sequence of methods used. |
| **Systematic data analysis described** | Authors provide a detailed procedure of analysis, with justification for the method of analysis |
| **Multivariate analysis used** | Authors report use of multivariate analysis to control for confounding |
| **Minimization of bias discussed** | Authors report steps taken to reduce measurement, social desirability, recall, and measurement biases. |
| **Integration of QUAN/QUAL components** | Authors report the integration of QUAN/QUAL methods and results |
| **Findings discussed** | Authors report the findings/results in terms of their impact on further research, programming and policy |

**References**

1. Hill J, Hoyt J, van Eijk AM, D'Mello-Guyett L, Ter Kuile FO, et al. (2013) Factors affecting the delivery, access, and use of interventions to prevent malaria in pregnancy in sub-Saharan Africa: a systematic review and meta-analysis. PLoS Med 10: e1001488.

2. O'Cathain A, Murphy E, Nicholl J (2008) The quality of mixed methods studies in health services research. Journal of Health Services Research & Policy 13: 92-98.

3. Pluye P, Gagnon MP, Griffiths F, Johnson-Lafleur J (2009) A scoring system for appraising mixed methods research, and concomitantly appraising qualitative, quantitative and mixed methods primary studies in Mixed Studies Reviews. International Journal Of Nursing Studies 46: 529-546.

4. Sale JEM, Brazil K (2004) A strategy to identify critical appraisal criteria for primary mixed-method studies. Quality & Quantity 38: 351-365.

5. Kiningu D.K. (2013) Factors influencing the use of evidence based guidelines in the management of malaria in pregnancy among health workers at Garissa provincial hospital, Kenya [Master’s thesis]. Nairobi: School of Public Health, University of Nairobi. Available: http://erepository.uonbi.ac.ke:8080/xmlui/bitstream/handle/11295/59598/Factors%20Influencing%20The%20Use%20Of%20Evidence%20Based%20Guidelines%20in%20the%20Management%20of%20Malaria%20in%20Pregnancy%20Among%20Health%20Workers%20at%20Garissa%20Provincial%20Hospital%20Kenya.pdf?sequence=3. Accessed 7 July 2014

6. Luz TC, Suarez-Mutis C, Miranda S, Moritz F, Freitas F, et al. (2013 B) Uncomplicated malaria among pregnant women in the Brazilian Amazon: Local barriers to prompt and effective case management. Acta Tropica 125: 137-142.

7. Sabin LL, Rizal A, Brooks MI, Singh MP, Tuchman J, et al. (2010) Attitudes, knowledge, and practices regarding malaria prevention and treatment among pregnant women in Eastern India. American Journal of Tropical Medicine and Hygiene 82: 1010-1016.

8. Smith Paintain L, Antwi GD, Jones C, Amoako E, Adjei RO, et al. (2011) Intermittent Screening and Treatment versus Intermittent Preventive Treatment of Malaria in Pregnancy: Provider Knowledge and Acceptability. Plos One 6: e24035.

9. Stangeland T, Alele PE, Katuura E, Lye KA (2011) Plants used to treat malaria in Nyakayojo sub-county, western Uganda. Journal of Ethnopharmacology 137: 154-166.

10. Tawfik L (2006) Mosquitoes, Malaria and Malarine: A Qualitative Study on Malaria Drug Use in Cambodia. Arlington, Virginia, USA: U. S. Agency for International Development.
