# Supplementary material for: Women's Access and Provider Practices for the Case Management of Malaria during Pregnancy: A Systematic Review and Meta-Analysis
Source: PLoS Med. 2014 Aug 5;11(8):e1001688. doi: 10.1371/journal.pmed.1001688 (PMC4122360; doi:10.1371/journal.pmed.1001688)
Supplement: Table S5 — Sub-group analysis for source of treatment among pregnant women. (DOCX) [file pmed.1001688.s005.docx]

Table S5. Sub-group analysis for source of treatment among pregnant women.

|  | N* | Pooled effect estimate (95% CI) | N | Pooled effect estimate (95% CI) | p-value† |
| --- | --- | --- | --- | --- | --- |
| **Type of question** | PW with fever | | If PW would have fever | |  |
| Doctor | 3 | 0.84 (0.65-0.94) | 1 | 0.82 (0.75-0.87) | 0.7 |
| Health facility or ANC | 4 | 0.60 (0.33-0.82) | 2 | 0.46 (0.01-0.98) | 0.8 |
| Self-Medication | 5 | 0.14 (0.06-0.32) | 2 | 0.08 (0.06-0.12) | 0.3 |
| Traditional healer/herbs | 5 | 0.03 (0.01-0.17) | 2 | 0.24 (0.01-0.93) | 0.3 |
|  |  |  |  |  |  |
| **Site of enrolment PW** | Antenatal clinic | | Community | |  |
| Doctor | 3 | 0.84 (0.65-0.94) | 1 | 0.82 (0.75-0.87) | 0.7 |
| Health facility or ANC | 3 | 0.70 (0.30-0.92) | 3 | 0.40 (0.10-0.80) | 0.3 |
| Self-Medication | 6 | 0.13 (0.05-0.27) | 1 | 0.10 (0.06-0.15) | 0.5 |
| Traditional healer/herbs | 5 | 0.05 (0.01-0.20) | 2 | 0.06 (0.0-0.99) | 1.0 |
|  |  |  |  |  |  |
| **Country of study** | Nigeria | | Not Nigeria | |  |
| Doctor | 2 | 0.90 (0.75-0.96) | 2 | 0.74 (0.53-0.88) | 0.1 |
| Health facility or ANC | 2 | 0.62 (0.26-0.88) | 4 | 0.52 (0.17-0.85) | 0.7 |
| Self-Medication | 3 | 0.09 (0.03-0.25) | 4 | 0.16 (0.05-0.40) | 0.5 |
| Traditional healer/herbs | 3 | 0.04 (0.00-0.78) | 4 | 0.08 (0.02-0.27) | 0.8 |
|  |  |  |  |  |  |
| **Location of residence** | Rural | | Urban | |  |
| Doctor | 2 | 0.74 (0.53-0.88) | 2 | 0.90 (0.75-0.96) | 0.1 |
| Health facility or ANC | 4 | 0.38 (0.14-0.70) | 2 | 0.84 (0.71-0.91) | 0.006 |
| Self-Medication | 3 | 0.20 (0.06-0.51) | 4 | 0.08 (0.04-0.19) | 0.2 |
| Traditional healer/herbs | 4 | 0.21 (0.06-0.52) | 3 | 0.02 (0.00-0.07) | 0.008 |

“Private clinic” and “Retail/pharmacy” not examined; only 1 and 3 studies in total, respectively

*N: indicates number of studies in subgroup

†Sub-group analysis was conducted with the program “Comprehensive Meta-Analysis”
