# Supplementary material for: Women's Access and Provider Practices for the Case Management of Malaria during Pregnancy: A Systematic Review and Meta-Analysis
Source: PLoS Med. 2014 Aug 5;11(8):e1001688. doi: 10.1371/journal.pmed.1001688 (PMC4122360; doi:10.1371/journal.pmed.1001688)
Supplement: Table S6 — Sub-group analysis for adherence to treatment policy among health care providers. (DOCX) [file pmed.1001688.s006.docx]

Table S6. Sub-group analysis for adherence to treatment policy among health care providers.

| Factor | N* | Pooled effect estimate (95% CI) | p-value† |
| --- | --- | --- | --- |
| **Trimester** |  |  |  |
| First trimester | 9 | 0.45 (0.19-0.75) | 0.067 |
| Other trimesters | 5 | 0.28 (0.14-0.47) |  |
| Trimester not specified | 7 | 0.72 (0.39-0.91) |  |
|  |  |  |  |
| First trimester | 9 | 0.28 (0.14-0.47) | 0.021 |
| Other trimesters | 5 | 0.72 (0.39-0.91) |  |
|  |  |  |  |
| **Level of prescriber** |  |  |  |
| Doctors only | 3 | 0.11 (0.04-0.23) | <0.001 |
| Other staff or mixed cadres | 18 | 0.52 (0.35-0.67) |  |
|  |  |  |  |
| **Method of study** |  |  |  |
| Self-administered questionnaires | 6 | 0.14 (0.07-0.28) | 0.001 |
| Interviews | 7 | 0.50 (0.27-0.73) |  |
| Record reviews | 8 | 0.66 (0.39-0.86) |  |
|  |  |  |  |
| **Country of study** |  |  |  |
| Nigeria | 10 | 0.25 (0.12-0.46) | 0.018 |
| Not Nigeria | 11 | 0.58 (0.40-0.75) |  |

*N: indicates number of studies in subgroup

†Sub-group analysis was conducted with the program “Comprehensive Meta-Analysis”
